# Investigating the efficacy of bioactive compounds from selected plant extracts against *Gibberella fujikuroi* species complex associated with damping off disease in sweet corn

**DOI:** 10.1038/s41598-025-05979-x

**Published:** 2025-07-01

**Authors:** Alyaa Abd Ali, Ayoob Obaid Alfalahi, Aalaa Khudhair Hassan, Ahlam Khalofah, Eilyn Mena, Abdelfattah A. Dababat, Fouad Mokrini

**Affiliations:** 1 Department of Plant Protection, College of Agriculture, University of Anbar, Anbar, Iraq; 2 Department of Plant Protection, College of Agricultural and Engineering Sciences, University of Baghdad, Baghdad, Iraq; 3 Department of Biology, College of Science, King Khalid University, Abha, Saudi Arabia; 4 Instituto de Investigaciones Biológicas Clemente Estable, Montevideo, Uruguay; 5 International Maize and Wheat Improvement Center (CIMMYT), Ankara, Turkey; 6 Nematology Laboratory, Biotechnology Research Unit, Regional Center of Agricultural Research, INRA, Rabat, Morocco

**Keywords:** Gibberella fujikuroi, Sweet corn, Phytoconstituents, Antifungal properties, In Silico, Biotechnology, Plant sciences

## Abstract

*Fusarium* genera are widespread disease-causing fungi that severely reduce plant productivity and yield quality, particularly in corn. In this study, we investigated the antifungal potential of selected plant extracts against damping-off disease-associated fungi in sweet corn (*Zea mays* L. saccharata). Fourteen *Fusarium* isolates were obtained from symptomatic sweet corn plants belonging to five different species, *viz. F. fujikuroi*, *F. proliferatum*, *F. verticillioides*, *F. oxysporum*, and *F. acuminatum*. Although all the isolated fungi were pathogenic, *F. verticillioides* (Fv-A), *F. fujikuroi* (Ff-A) and *F. oxysporum* (Fo-W2) were more aggressive showing higher values for infection (%) and infection severity (%) and negatively affected seed germination (%) and other growth variables. Phytochemical analysis for five wildly growing plant species namely; *Eruca vesicaria* L., *Strigosella africana* L., *Chenopodium album* L., *Oxalis pes-caprae* L. and *Ducrosia ismaelis* was conducted using GC-MS analysis. The most abundant bioactive compounds in the three selected extracts (*E. vesicaria*,* O. pes-caprae* L. and *D. ismaelis*) were 1-eicosanol, (Z)6,(Z)9-Pentadecadien-1-ol and n-Hexadecanoic acid, and Nonadecane, respectively. Oxacyclotricosan-2-one and D-Homoandrostane from *E. vesicaria* L.; Vitamin E and Benzenepropanoic acid, 3,5-bis(1,1-dimethylethyl)-4-hydroxy-, methyl ester from *O. pes-caprae* L. and 7.beta.-(1-hydroxy-1-methylethyl), alpha-gurjunene from *D. ismaelis* showed extraordinary molecular docking and dynamic properties including high binding free energy, relatively low inhibition constant (pKi), ligand efficiency, and low torsional energy against three fungal enzymes, namely GH10 xylanase, Plant-type chitinase inhibitors, and Sterol 14-alpha Demethylase. Thus, these bioactive compounds can be listed as potential binders of these target proteins and could be used in designing new fungicides.

**Supplementary Information:**

The online version contains supplementary material available at 10.1038/s41598-025-05979-x.

## Introduction

The detrimental impact of fungi-related diseases is due to their destructive effects on agricultural crops, and humans have experienced this effect on several occasions around the world, where land degradation, economic destruction, and famine have mostly been caused by fungal diseases^1^.

In Iraq, the utilization of sweet corn is growing dramatically; thus, cultivation areas have expanded accordingly. Locally, the corn cultivated area amounts to 100,000 ha with an estimated production of 500,000 tons during 2023^2^. The expansion of sweet corn-planted areas is accompanied by the incidence of new diseases due to novel pathogens or altered pathogen preference, which allows pathogens to develop new strategies to spread and infect new host plants^2^. In addition, pathogenic fungi with a broad host range, such as Fusarium, may have a number of alternative plant hosts. The collective host range of some pathogens enable them to passage through alternative hosts that considerably alters aggressiveness and ultimate fitness of pathogenic fungi^3^.

Sweet corn (*Zea mays* L. *saccharata*) is a commodity crop because of its high sugar content, commercial potential, and unfortunate vulnerability to fungal diseases. These fungi cause various diseases mainly root rot and damping-off, which compromise the production and marketing value of sweet corn^4^.

*Fusarium* spp. occupy an important position regarding variety, damage, dissemination, and economic losses compared with other soil-borne pathogenic fungi. Being ubiquitous, Fusarium infects nearly all economically important crops, in addition to humans and their animals; hence, it is known as a trans-kingdom pathogen^5,6^. The *Gibberella fujikuroi* species complex is a common *Fusarium* genus that infects aerial or underground plant parts; as a primary or secondary source of infection; and may be saprotrophic or hemibiotrophic, causing wilting, rotting, and seedling death diseases^7^.

Synthetic fungicides have been widely applied to manage phytopathogens because of their quick and strong effect; however, the extensive use of chemical fungicides is of great concern and seriously threatens the food chain and earth’s environment. Therefore, new and more sustainable alternatives are urgently required to control plant diseases^8^. Plant extracts, specifically those from wild plants, are natural products with potential antifungal activities. They have attracted increasing attention because of their availability, applicability, affordability, and eco-friendly characteristics^9^. The typical presence of different bioactive compounds in combinations gives the plant extracts an advantage over the use of pure compounds in their synergistic mechanism of action, especially those marked with antimicrobial properties. Recently reported results confirmed the antimicrobial effects of several wildly growing plant types including *Eruca vesicaria* L.^10^, *Strigosella africana* L.^11^, *Chenopodium album* L.^12^, *Oxalis pes-caprae* L.^13^ and *Ducrosia ismaelis*^14^.

Continued progress in biotechnology and bioinformatics has substantially contributed to the development of environmentally independent and precise tools for studying and diagnosing genetic variation among phytopathogens. This makes it possible to monitor how genetic variation and crop management affect host plant performance at the cellular and molecular levels, thereby providing more accurate decisions^15^. Molecular docking is an emerging computational technique that employs computer software alongside chemical structure information to predict the interaction between the ligand and target protein^16,17^. Therefore, this study was designed to detect damping-off associated fungal pathogens in sweet corn (*Zea mays* L. *saccharata*) and investigate the antifungal properties of natural plant extracts against the companion pathogenic fungi in silico, in vitro, and *in planta*.

## Results

### Phenotypic and molecular identification of pathogenic fungi

The phenotypic identification of the isolated fungal phytopathogens was based on colony color, hypha growth, and conidial macro- and microscopic characteristics (Supplementary Fig. S1). A total of 14 fungal isolates were recovered from the roots and stem bases of infected sweet corn plants, which were distributed among five different species of Fusarium, namely, *fujikuroi* (one isolate), *proliferatum* (two isolates), *verticillioides* (eight isolates), oxysporum (two isolates), and acuminatum (one isolate). These results were further confirmed by sequencing the Internal Transcribed Spacer (ITS) of the rDNA and Translational Elongation Factor 1-a (TEF1-a) regions. The ITS region was used for general fungal identification, while TEF1-α provided higher resolution at the species level within Fusarium, which successfully amplified approximately 650 bp and 700 bp DNA fragments, respectively. Fourteen ITS and three TEF1-a sequences of the phenotypically identified Fusarium isolates were deposited in the National Center for Biotechnology Information (NCBI) (http://www.ncbi.nlm.nih.gov) under deposition numbers LC807017, LC807018, LC807019, LC807021, LC807022, LC807023, LC807024, LC807025, LC807026, LC807027, LC807028, LC807029, LC807031, and LC807032 for ITS sequences; and PV471465, PV471466 and PV471467 for TEF1-a sequences, respectively (Supplementary Table S1).

### In vitro assessment of pathogenicity

**Fig. 1:**
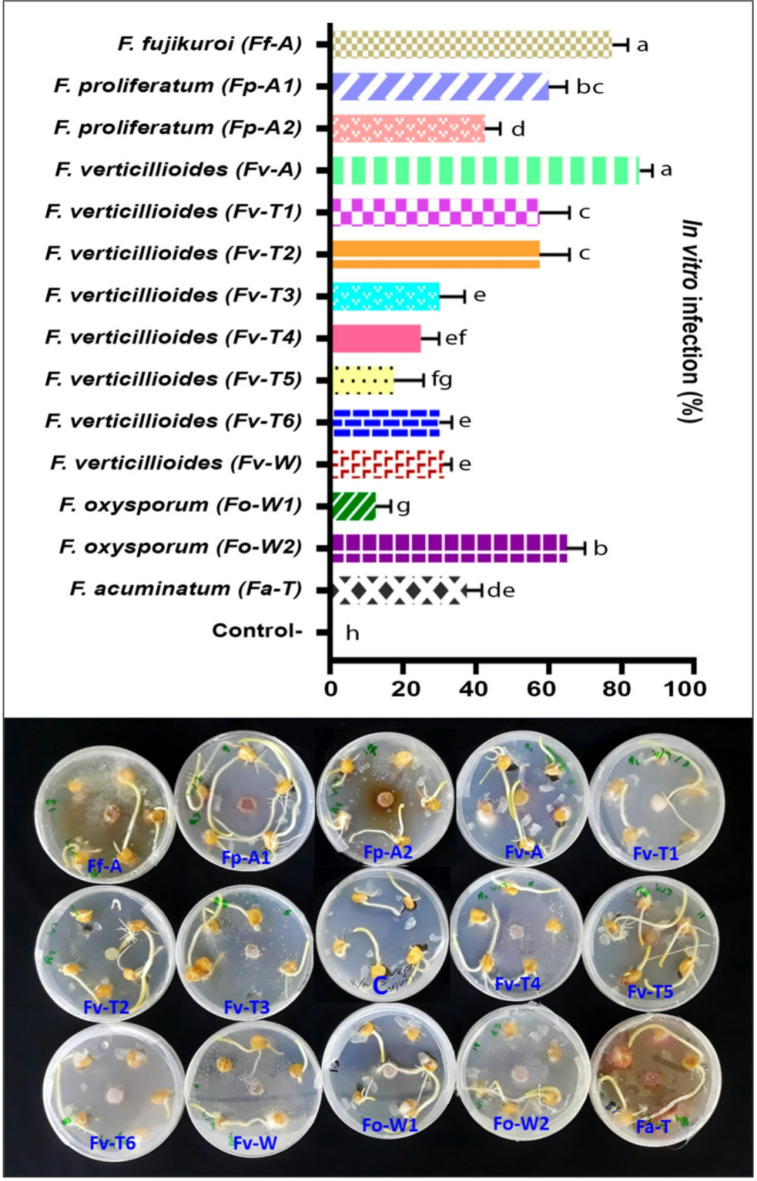
In vitro assessment of the pathogenicity of sweet corn seeds grown on PDA medium. Ff-A (*Fusarium fujikuroi*), Fp-A1 (*Fusarium proliferatum*), Fp-A2 (*Fusarium proliferatum*), Fv-A (*Fusarium verticillioides*), Fv-T1 (*Fusarium verticillioides*), Fv-T2 (*Fusarium verticillioides*), Fv-T3 (*Fusarium verticillioides*), Fv-T4 (*Fusarium verticillioides*), Fv-T5 (*Fusarium verticillioides*), Fv-T6 (*Fusarium verticillioides*), Fv-W (*Fusarium verticillioides*), Fo-W1 (*Fusarium oxysporum*), Fo-W2 (*Fusarium oxysporum*), Fa-T (*Fusarium acuminatum*) and C (control-). The error bars represent the standard error of the difference. Different letters above error bars indicate statistically significant differences (*P ≤ 0.05*).

The results of the in vitro assessment of pathogenicity clearly showed that all tested isolates were pathogenic to sweet corn (Fig. 1). Each fungal isolate was re-isolated from infected plants to confirm Koch’s postulates, while no fungi were recovered from the control plants. However, the pathogenicity differed significantly (*P ≤ 0.05*), indicating a wide range of infection capabilities. The highest rates of infection were 85%, 77.5%, and 65% for *F. verticillioides* (Fv-A), *F. fujikuroi* (Ff-A), and *F. oxysporum* (Fo-W2), respectively; however, a weak infection (12.5%) was observed for the *F. oxysporum* isolate (Fo-W2). On the other hand, a higher level of pathogenicity was detected in some other distant fungal isolates, such as *F. fujikuroi* (Ff-A), *F. oxysporum* (Fo-W2), and *F. proliferatum* (Fp-A1), which were 77.5%, 65%, and 60%, respectively (Fig. 1). Notably, most of the identified isolates were *F. verticillioides*, indicating a high frequency of this species of Fusarium genera.

### In planta assessment of pathogenicity

**Fig. 2:**
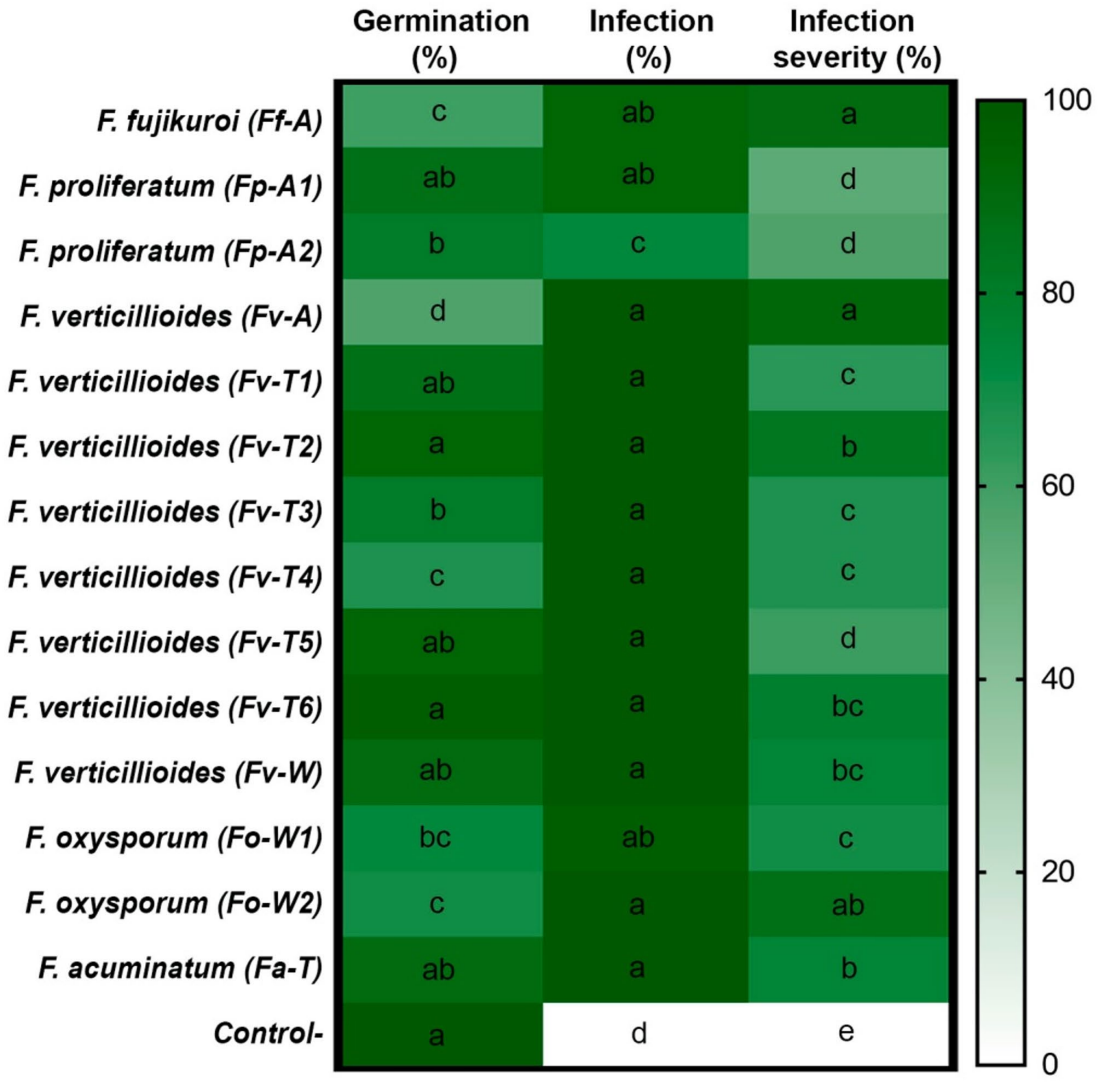
Heatmap of *in planta* assessment of pathogenicity of 14 fungal isolates from sweet corn plants. Different letters indicate statistically significant differences (*P* ≤ 0.05).

The *in planta* assessment of pathogenicity was conducted in a pot experiment, in which significant differences (*P* ≤ 0.05) between the pathogenic fungi in terms of the percentage of germination, infection, and infection severity were detected (Fig. 2). Regarding germination percentage, the pathogenic fungi had a significant effect on the germinated seeds of sweet corn, even between different isolates of the same species, ranging from 56.67 to 93.33% for *F. verticillioides* isolates Fv-A and Fv-T2, respectively. *F. fujikuroi* (Ff-A) showed a higher ability to infect sweet corn seeds and negatively affected seed viability resulting in 60% germination percentage compared to the control groups that showed 100% seed germination.

In general, the high significant infection values referred to the aggressive attitude of most of the tested fungal isolates, but *F. verticillioides* isolates were superior, reflecting a 100% infection percentage. In contrast, *F. proliferatum* acted differentially, resulting in a minimal infection percentage (73.33%).

Significant differences in infection severity (%) were detected between the 14 fungal isolates, with values ranging between higher values of 91.67%, 90.00%, and 86.67% for *F. verticillioides* (Fv-A), *F. fujikuroi* (Ff-A) and *F. oxysporum* (Fo-W2), respectively. In contrast, only two isolates of *F. proliferatum* (Fp-A1 and Fp-A2) were less aggressive, with infection severities of 52.67% and 56.67%, respectively. The control group had a 0% infection severity (Fig. 2).

### In vitro assessment of plant extracts antagonistic effect

#### Plant extracts antagonistic effect against *F. fujikuroi*

**Fig. 3:**
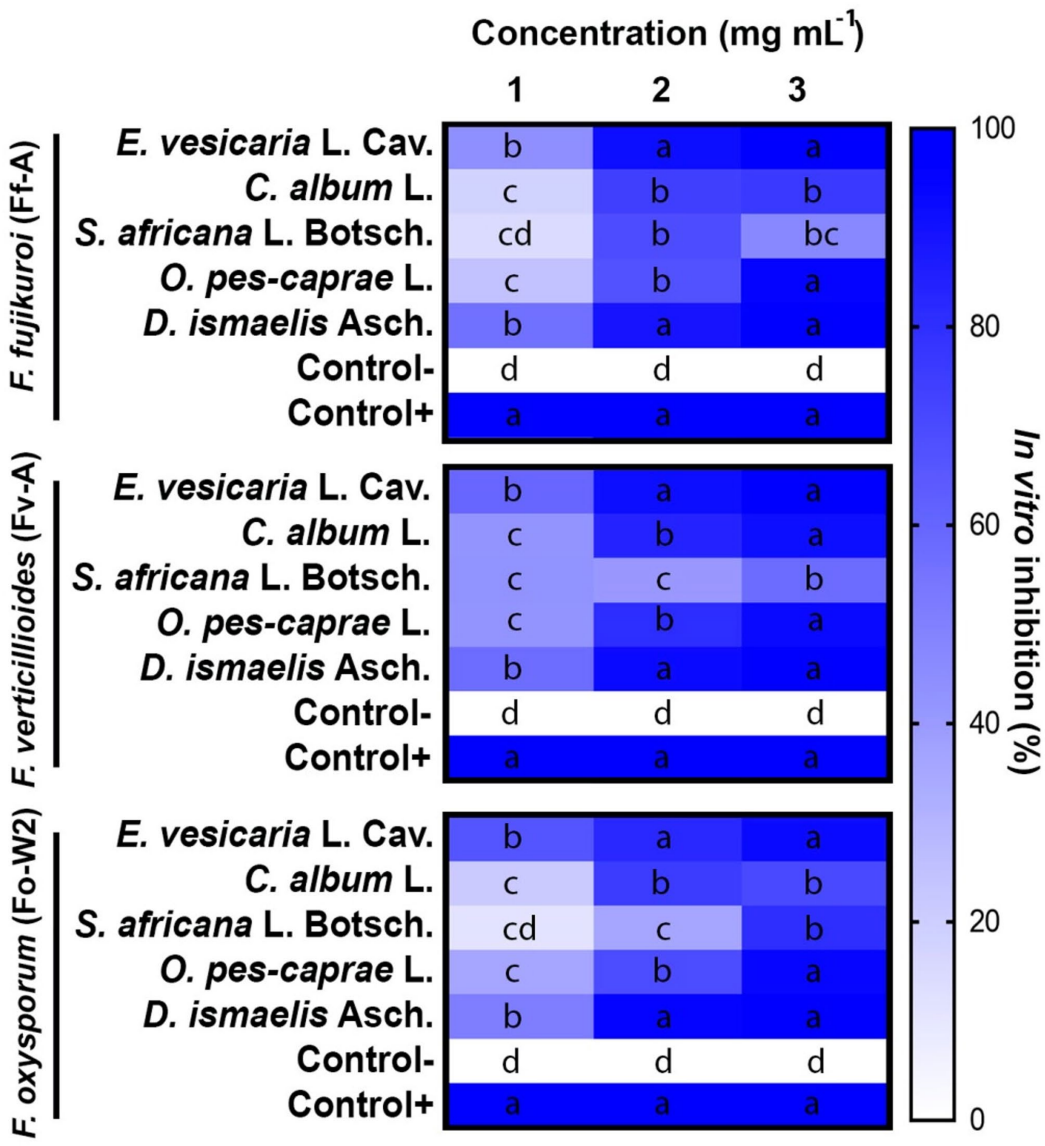
Heatmap of in vitro antagonistic assessment of the five plant extracts (*E. vesicaria* L. Cav., *C. album* L., *S. africana* L. Botsch, *O. pes-caprae* L., and *D. ismaelis* Asch.) at three concentrations (1, 2, and 3 mg L^− 1^) against three fungal isolates (*F. fujikuroi* (Ff-A), *F. verticillioides* (Fv-A) and *F. oxysporum* (Fo-W2)). Different letters indicate statistically significant differences (*P* ≤ 0.05).

The antagonistic effect of the five plant extracts namely, Arugula (*E. vesicaria* L. Cav.), white goosefoot (*C. album* L.), African mustard (*S. africana* L. Botsch.), Bermuda buttercup (*O. pes-caprae* L.), and Ducrosia (*D. ismaelis* Asch.) was investigated at three concentrations (1 mg mL^− 1^, 2 mg mL^− 1^ and 3 mg mL^− 1^) against the three selected fungal isolates with the highest pathogenicity under in vitro and *in planta* conditions (Fig. 3).

The five plant extracts showed significantly different abilities (*P* ≤ 0.05) to inhibit *F. fujikuroi* growth; however, *D. ismaelis* showed the highest inhibitory effect against this fungal isolate (81.22%). In contrast, *S. africana* had the lowest inhibitory effect, with only a 43.33% inhibitory percentage, compared to the negative and positive control groups that showed 0% and 100% inhibitory percentage, respectively (Fig. 3).

The three applied concentrations were also significantly different (*P* ≤ 0.05), with the highest concentration (3 mg mL^− 1^) hindering *F. fujikuroi* development by 73.67%. The minimum concentration (1 mg mL^− 1^) resulted in only 36.57% inhibition rate, which was the lowest compared to the other two concentrations. Significant interactions were observed between the five plant extracts and their corresponding concentrations (*P ≤ 0.05*). The interaction ranged between 99.33% for *D. ismaelis* and *E. vesicaria*, and 14.33% for *S. africana* (Fig. 3).

#### Plant extracts antagonistic effect against *F. verticillioides*

The obtained results of the antagonistic analysis showed significant antifungal activity against *F. verticillioides*, depending on the plant extract and concentration applied (*P* ≤ 0.05). Maximal inhibition was caused by *E. vesicaria* (83.56%), followed by *D. ismaelis* (81.78%), whereas *S. africana* was less effective when it exhibited the lowest antagonistic effect (47%).

Among the three applied concentration treatments, the 3 mg mL^− 1^ treatment was superior, with 76.71% inhibition. The lowest concentration of 1 mg mL^− 1^ resulted in 49.10% inhibition (Fig. 3).

The superiority of *E. vesicaria* at 3 mg mL^− 1^ (100%) was not different from that of the positive control, where fungicide was included. *O. pes-caprae* followed by *C. album* acting differently in response to the lowest concentration (1 mg mL^− 1^) by showing a minimum of 42% and 42.33%, respectively (Fig. 3).

#### Plant extracts antagonistic effect against *F. oxysporum*

The application of five ethanomethanolic extracts at three different concentrations effectively controlled the mycelial growth of *F. oxysporum*. The results demonstrated that the inhibition rate varied considerably depending on the applied extract and its concentration. For plant extracts, *D. ismaelis* and *E. vesicaria* displayed the highest inhibitory rates of 81.56% and 80.11%, respectively. In contrast, *S. africana* showed modest antifungal activity (Fig. 3).

The results revealed that the applied concentrations had significantly varied inhibitory effects ranging between 76.48% and 40.71% for 3 mg mL^− 1^ and 1 mg mL^− 1^, respectively.

 Fig. 3, indicates a significant interaction between plant extracts and concentrations, in which the interaction values ranged between 99.33% and 11.33% for *D. ismaelis* at 3 mg mL^− 1^ and *S. africana* at 1 mg mL^− 1^. The negative and positive controls showed 0% and 100% inhibition rates, respectively.

### In planta antagonistic assessment of selected plant extracts

#### Seed germination (%)

According to the results of the in vitro assessment of the antagonistic effect of plant extracts, the antifungal activity of the three selected plant extracts, namely *E. vesicaria* L. Cav., *O. pes-caprae* L., and *D. ismaelis* Asch. was examined against three selected pathogenic fungi that revealed the highest values of infection and infection severity during in vitro and in planta assessments, namely *F. fujikuroi* (Ff-A), *F. verticillioides* (Fv-A), and *F. oxysporum* (Fo-W2).

The selected fungal isolates had a significant negative effect on seed germination in the pot experiment. Although *F. verticillioides* reduced seed germination the most (66.67%), the *F. oxysporum* was found to be less effective in reducing seed germination to 72%.

**Fig. 4:**
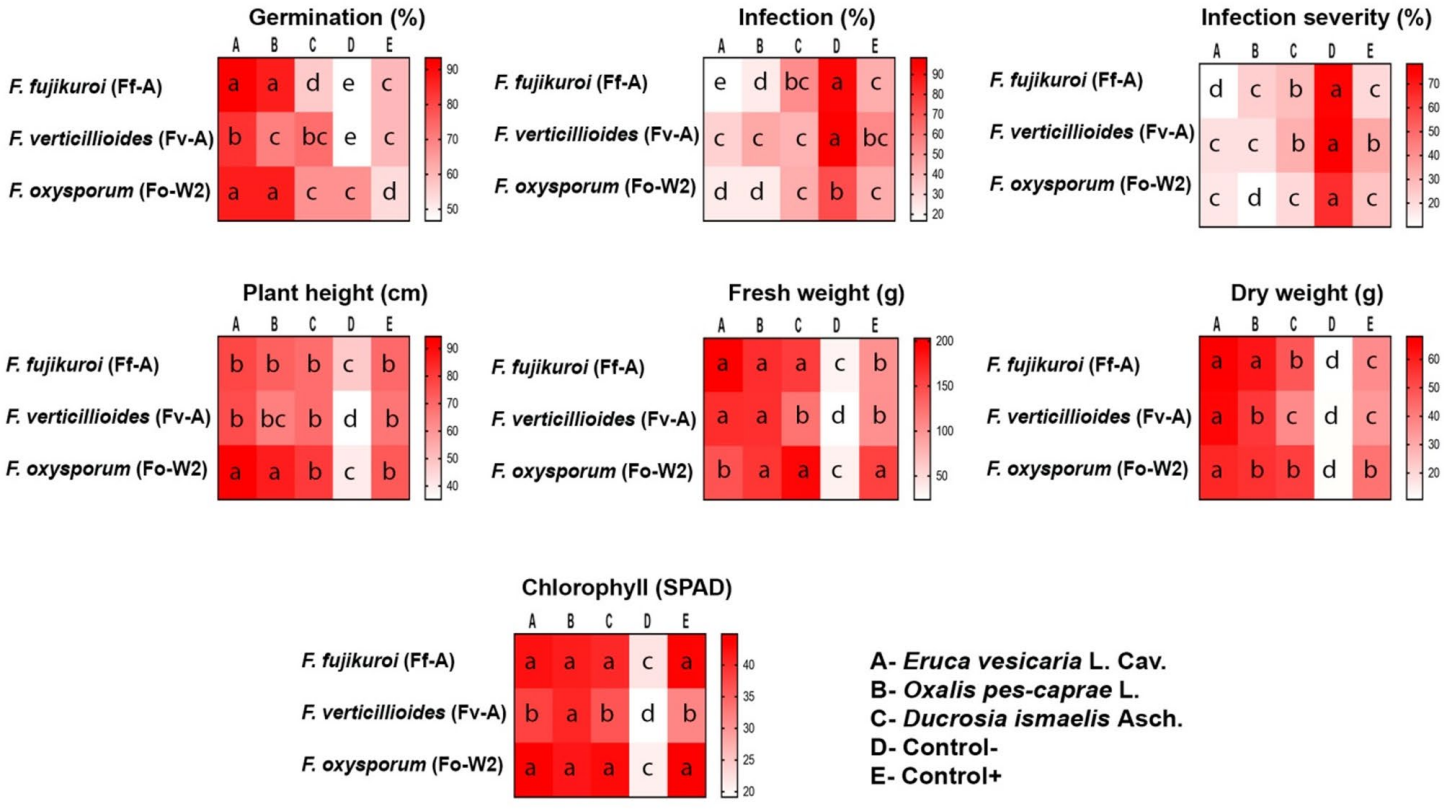
Heatmap of the effect of pathogenic fungi (*F. fujikuroi* (Ff-A), *F. verticillioides* (Fv-A) and *F. oxysporum* (Fo-W2) and plant extracts (*E. vesicaria* L. Cav., *O. pes-caprae* L. and *D. ismaelis* Asch.) on the Germination (%), Infection (%), Infection severity (%), Plant height (cm), Fresh weight (g), Dry weight (g), and Chlorophyll (SPAD). Different letters indicate statistically significant differences (*P* ≤ 0.05).

All the applied plant extracts significantly affected seed germination; among these, *D. ismaelis* demonstrated the highest effect (55.56%), followed by *O. pes-caprae* (81.11%), and *E. vesicaria* (87.78%). The negative and positive control groups showed modest efficiency in controlling pathogenic infection, with 53.33% and 57.78% germination rates, respectively (Fig. 4).

#### Infection (%)

The results of the statistical analysis of the percentage of infection affected by pathogenic fungi and plant extracts are presented in Fig. 4. These results indicate a clear significant effect of both the studied factors, fungal isolates, and plant extracts. However, *F. verticillioides* was more aggressive than the other two Fusarium isolates, exhibiting the highest infection rate of 48.3%, followed by *F. fujikuroi*, and *F. oxysporum* (40% and 38%, respectively).

In terms of different plant extracts, the maximum inhibition was recorded for *E. vesicaria*, which resulted in the lowest infection rate (23.9%). In contrast, *D. ismaelis* was less effective in reducing the infection rate, with the highest recorded infection rate of 46.1%. For the control groups, the negative control showed a maximum infection rate of 88.30%, while a 47.2% infection rate was reported for the positive control (Fig. 4).

The two-way interaction between the *E. vesicaria* plant extract and *F. fujikuroi* fungal isolate exhibited the lowest significant infection rate of 16.7%. In the same context, *D. ismaelis* showed modest activity against the same fungus, with 53.3% infection compared to the control groups, which showed higher values.

#### Infection severity (%)

Among the tested fungal isolates, *F. verticillioides* exhibited a noticeable infection severity of 39.99%, while lower infection severity was detected in *F. fujikuroi* and *F. oxysporum* (35.33% and 31.67%, respectively) (Fig. 4).

The results highlighted a significant reduction in the severity of infection of the tested fungi in response to the applied extracts. However, the superiority of *E. vesicaria* resulted in the lowest infection severity (23.9%). In contrast, *D. ismaelis* was less effective at reducing the infection rate, with the highest recorded value (46.1%) compared to 72.78% and 26.67% for negative and positive control groups, respectively.

As shown in Fig. 4, the significant two-way interaction between the different levels of the two studied factors ranged between 10% and 31.67% for *F. fujikuroi* and *F. verticillioides* isolates in response to *E. vesicaria* and *D. ismaelis* treatments, respectively.

#### Plant height (cm)

Based on the analysis of variance results, all the investigated pathogenic fungi had significant effects on plant height. ranged between 74.76 cm and 62.43 cm for *F. fujikuroi* and *F. verticillioides*, respectively (Fig. 4).

Evaluation of plant extract efficacy revealed significant antifungal activity of the three ethanomethanolic extracts tested in the pot experiment. The highest mean plant height was achieved by the *E. vesicaria* treatment (82.69 cm), in contrast to 72.53 cm plant height shown by *D. ismaelis* extract. All treatments differed significantly compared to negative and positive groups (40.99 cm and 70.17 cm, respectively).

The selected three fugal isolates respond differentially to the applied plant extracts, however, *F. oxysporum* produced 94.33 cm plant height in response to *E. vesicaria* treatment while *F. verticillioides* produced 64.87 cm in response to *O. pes-caprae* treatment (Fig. 4).

#### Fresh weight (g)

The data showed a significant effect of the tested fungal isolates on fresh weight of sweet corn plants. The maximum fresh weight was observed for *F. oxysporum* at 155.9 g. Contrarily, *F. verticillioides* and *F. fujikuroi* caused significant reduction in the fresh weight having almost the same mean values with 101.8 g and 101.7 g, respectively (Fig. 4).

Among all the different treatments of plant extracts, the best effect was exhibited by *E. vesicaria* treatment with 171.5 g fresh weight, compared to 158 g achieved by *D. ismaelis*. These treatments differed significantly between the negative (29.8 g) and positive (119.8 g) control groups (Fig. 4).

The two-way interaction ranged between high (203 g) and low (143.8 g) values for *F. fujikuroi* and *F. oxysporum* treated with *E. vesicaria* extract.

#### Dry weight (g)

Figure 4 presents the individual and combined effect of three selected pathogenic fungi besides three plant extracts. The data analysis revealed significant differences between the three fungal isolates in dry weight, which ranged from 42 g to 36.67 g for *F. oxysporum* and *F. fujikuroi*, respectively.

Among the three plant extracts, *E. vesicaria* was the most effective plant extract in reducing the negative effects of fungal isolates, resulting in the highest mean of dry weight 63.33 g, while only 46.22 g of dry weight was achieved by *D. ismaelis*. Contrarily, 119.8 g and 29.8 g were recorded for positive and negative control groups, respectively (Fig. 4).

Two-way interactions were significant, in which the examined fungal isolates produced 68 g and 37.33 g of dry weight in response to *E. vesicaria* and *D. ismaelis* extracts, respectively.

#### Chlorophyll content

The chlorophyll content showed significant differences according to the investigated pathogenic fungi and the applied extracts. Regarding fungal isolates, the highest value was observed for *F. oxysporum* (39.02), followed by *F. fujikuroi* (37.87), while only 33.05 was recorded for the *F. verticillioides* isolate (Fig. 4).

Among the three tested plant extracts, *E. vesicaria* and *O. pes-caprae* showed almost the same activity against the pathogenic fungi with 41.7 and 41.28 chlorophyll content, respectively. In contrast, the *D. ismaelis* extract showed a significantly lower effect, with 39.78. The control groups demonstrated relatively low (20.64) and high (39.83) chlorophyll content in the negative and positive controls, respectively.

Two-way interaction varied significantly regarding chlorophyll content, with values ranging between 44.87 for *F. oxysporum* affected by *E. vesicaria* and 36.17 for *F. verticillioides* affected by *D. ismaelis* (Fig. 4).

### Phytochemical analysis of ethanomethanolic extracts

Phytochemical analysis of the five examined plant extracts was performed using gas chromatography-mass spectrometry (GC-MS) technique (Supplementary Figs. S2–S6). The chemical profile of the ethanomethanolic extracts revealed the existence of 40 active components in each of *E. vesicaria* L., *C. album* L., and *D. ismaelis*. Nevertheless, *O. pes-caprae* L. had 36 active components, whereas *S. africana* L. had only 18 (Supplementary Table S2). The identified phytochemicals belong to different chemical categories, mainly fatty acids, alkanes, esters, terpenoids, flavonoids, glycosides, sterols, phenolics, saponins, and heterocyclic compounds. The detected chemical constituents were sorted according to the retention time (RT) and percentage area (%), as shown in Supplementary Table S2.

The *E. vesicaria* ethanomethanolic extract was predominated by 1-eicosanol (15%) followed by Heptacosane (8%), Docosanoic acid, ethyl ester (7.27%) and n-Tetracosanol-1 (7.01%). For *C. album* L. extract, two fatty acids were the major components, n-Hexadecanoic acid and Oleic acid, which had the highest areas of 18.25% and 16.25%, respectively. However, the ethanomethanolic extract of *D. ismaelis* Asch. elucidated the prevalence of nonadecane (24.44%), followed by heneicosane (9.87%) and 8-ISOPROPYL-1-METHYL-5-METHYLENE-1,6-CYCLODECADIENE (8.29%).

Thirty six compounds were identified from the ethanomethanolic extract of *O. pes-caprae* L. From the GC-MS profile, the most abundant components were three phytochemicals, namely, (Z)6,(Z)9-Pentadecadien-1-ol, n-Hexadecanoic acid, and Celidoniol, deoxy, as the percentage of the peak area reached 21.81%, 18.41%, and 12.07%, respectively (Supplementary Table S2).

Less chemical constituents (18) were determined from the ethanomethanolic extract of *S. africana* L., of which 1 H-Indene, 2-butyl-5-hexyloctahydro-, Nonacosane and n-Hexadecanoic acid were found in higher quantity according to the peak area that reached 20.47%, 6.62% and 6.13%, respectively (Supplementary Table S2).

### Molecular docking analysis

According to the GC-MS investigation and in vitro antagonistic assessment of plant extracts, three plant extracts of *E. vesicaria* L., *O. pes-caprae* L. and *D. ismaelis*. was subjected to further molecular docking investigations. The binding free energy (kcal/mol), inhibition constant pKi (µM), ligand efficiency (kcal/mol/non-H atom), and torsional energy between the phytochemicals of the three selected extracts (ligands) and three fungal enzymes, namely, GH10 xylanase (PDB ID: 3U7B), plant-type chitinase inhibitors (PDB ID: 4TXE), and Sterol 14-alpha Demethylase (PDB ID: 5FRB) were estimated (Supplementary Table S3).

**Fig. 5:**
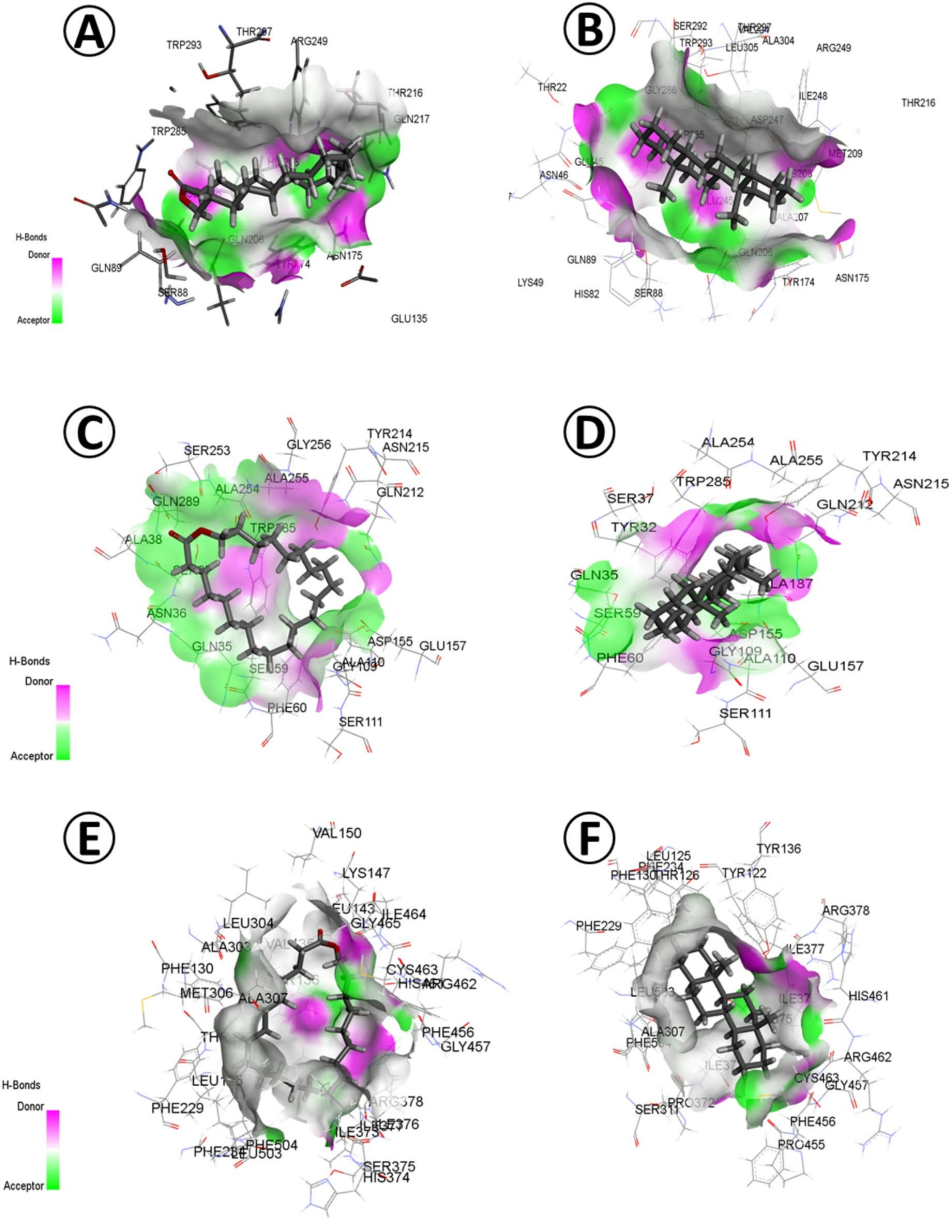
Molecular docking of *E. vesicaria* L. Cav. (**A**) Oxacyclotricosan-2-one and GH10 xylanase (PDB ID: 3U7B). (**B**) D-Homoandrostane (5. alpha.,13.alpha.) and GH10 xylanase. (**C**) Oxacyclotricosan-2-one and Plant-type chitinase inhibitors (PDB ID: 4TXE). (**D**) D Homoandrostane, (5.alpha.,13.alpha.) and Plant-type chitinase inhibitors. (**E**) Oxacyclotricosan-2-one and Sterol 14-alpha Demethylase (PDB ID: 5FRB). (**F**) D Homoandrostane, (5.alpha.,13.alpha.) and Sterol 14-alpha Demethylase.

Regarding *E. vesicaria* L. Cav. ethanomethanolic extract, the binding free energy was highest between GH10 xylanase and each D-homoandrostane (5. alpha.,13.alpha.), 14-. BETA.-H-PREGNA, Oxacyclotricosan-2-one, Culmorin and Cyclohexane, 1-(1,5-dimethylhexyl)-4-(4-methylpentyl) revealing − 9.7, -9, -8.8 and − 7.6 kcal/mol (Fig. 5).

Although plant-type chitinase inhibitors exhibited the highest binding free energy against almost the same phytochemicals, relatively low values were recorded for cyclohexane, 1-(1,5-dimethylhexyl)-4-(4-methylpentyl) that showed − 7.3 kcal/mol, followed by D-Homoandrostane, (5. alpha.,13.alpha.), Oxacyclotricosan-2-one, 14-.BETA.-H-PREGNA and 2,6,10,14-Tetramethyl-7-(3-methylpent-4-enylidene) pentadecane with − 7.3, − 7, − 6.8 and − 6.5 kcal/mol, respectively.

Oxacyclotricosan-2-one and D-Homoandrostane (5. alpha.,13.alpha.) exhibited low inhibition constant (pKi) and ligand efficiency against the targeted fungal enzyme GH10 xylanase (2.93 and 2.93, 0.3667 and 0.485 µM), while Plant-type chitinase inhibitors displayed a higher pKi values (5.13 and 5.35). Contrarily, Sterol 14-alpha Demethylase showed much higher pKi values against Oxacyclotricosan-2-one and D-Homoandrostane active components of *E. vesicaria* L. Cav. ethanomethanolic extract (7.26 and 6.82 µM, respectively). Torsional energy was zero for the two active components against the three fungal enzymes examined.

**Fig. 6:**
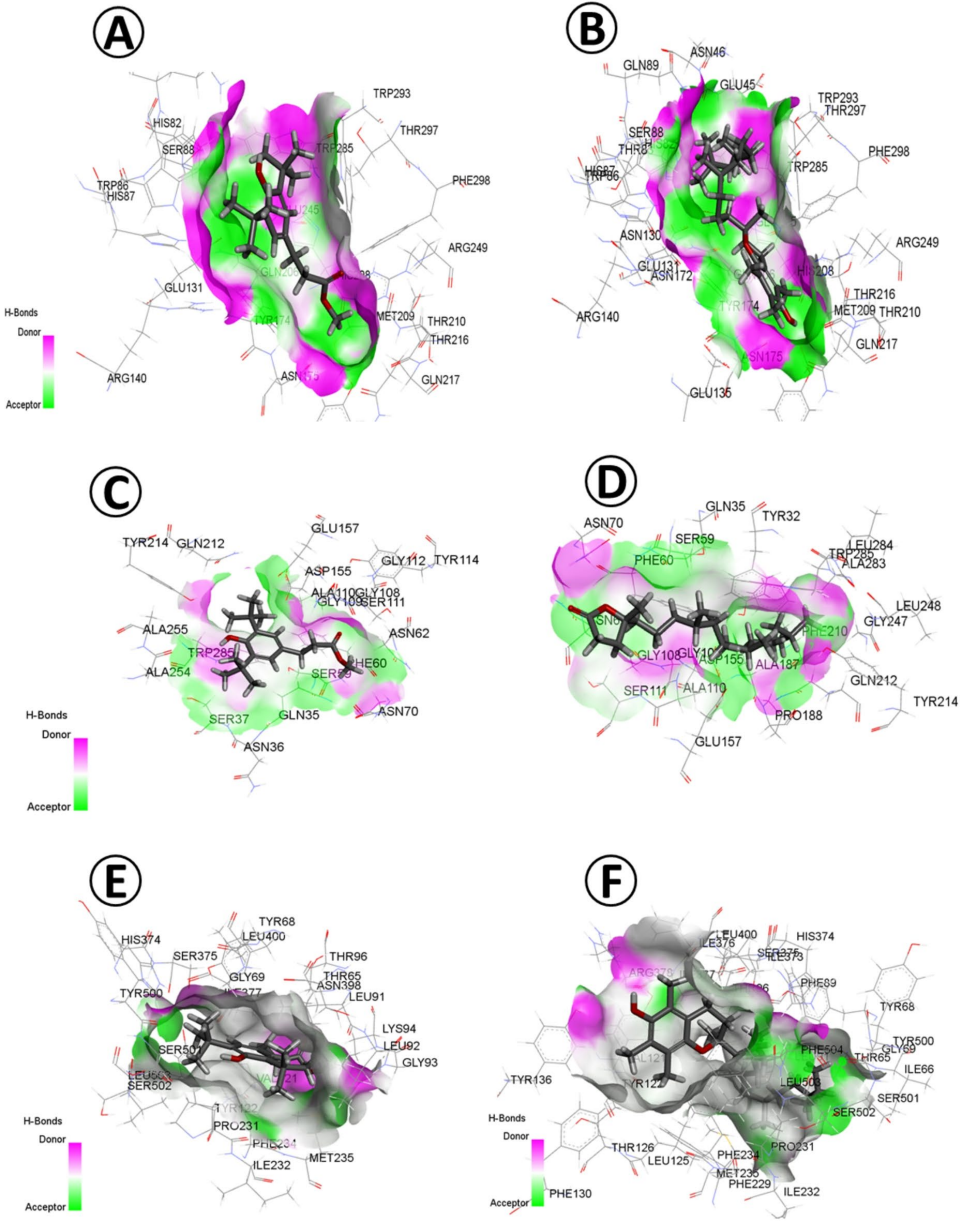
Molecular docking of *O. pes-caprae L.* (**A**) Benzenepropanoic acid, 3,5-bis(1,1-dimethylethyl)-4-hydroxy-, methyl ester, and GH10 xylanase (PDB ID: 3U7B). (**B**) Vitamin E and GH10 xylanase. (**C**) Benzenepropanoic acid, 3,5-bis(1,1-dimethylethyl)-4-hydroxy-, methyl ester and Plant-type chitinase inhibitors (PDB ID: 4TXE). (**D**) Vitamin E and Plant-type chitinase inhibitors. (**E**) Benzenepropanoic acid, 3,5-bis(1,1-dimethylethyl)-4-hydroxy-, methyl ester and Sterol 14-alpha Demethylase (PDB ID: 5FRB). (**F**) Vitamin E and Sterol 14-alpha Demethylase.

The 36 phytoconstituents of *O. pes-caprae* L. showed a wide range of docking parameters, of which binding free energy reached the highest values of − 7.5, − 7.2, − 6.9, − 6.5 and − 6.4 kcal/mol between GH10 xylanase target enzyme and each of Vitamin E, Benzenepentanoic acid, .delta.-hydroxy-.beta.-oxo-, methyl ester, Benzenepropanoic acid, 3,5-bis(1,1-dimethylethyl)-4-hydroxy-, methyl ester, 2-Hexadecen-1-ol, 3,7,11,15-tetramethyl-, [R-[R*,R*-(E)]]-, and Dibutyl phthalate, respectively (Fig. 6). Similarly, these ligands had the highest pKi values ranging between 5.5 and 4.84 µM, meanwhile moderately low ligand efficiency and torsional energy can be noticed (Supplementary Table S3).

Plant-type chitinase inhibitors displayed maximum binding free energy (kcal/mol) toward Benzenepropanoic acid, 3,5-bis(1,1-dimethylethyl)-4-hydroxy-, methyl ester (− 6.6), 4,8,12,16-Tetramethylheptadecan-4-olide (-6.5), Benzene (− 6.4), Benzenepentanoic acid (− 6.3), .delta.-hydroxy-.beta.-oxo-, methyl ester (− 6.3) and 9,12,15-Octadecatrienoic acid, (Z, Z,Z) (− 6.3). Also, the detected inhibitory constant (pKi) was in higher values for these ligands ranging between 4.84 µM for Benzenepropanoic acid, 3,5-bis(1,1-dimethylethyl)-4-hydroxy-, methyl ester and 4.62 µM for 9,12,15-Octadecatrienoic acid, (Z, Z,Z).

The Sterol 14-alpha Demethylase had higher docking values including binding free energy that reached − 9.2, − 8.4 kcal/mol for Vitamin E and Benzenepentanoic acid, respectively. These ligands were marked with the maximum inhibitory constant (6.75 and 6.16 µM, respectively). However, high to moderate ligand efficiency (kcal/mol/non-H atom) and torsional energy values were indicated (Supplementary Table S3).

**Fig. 7:**
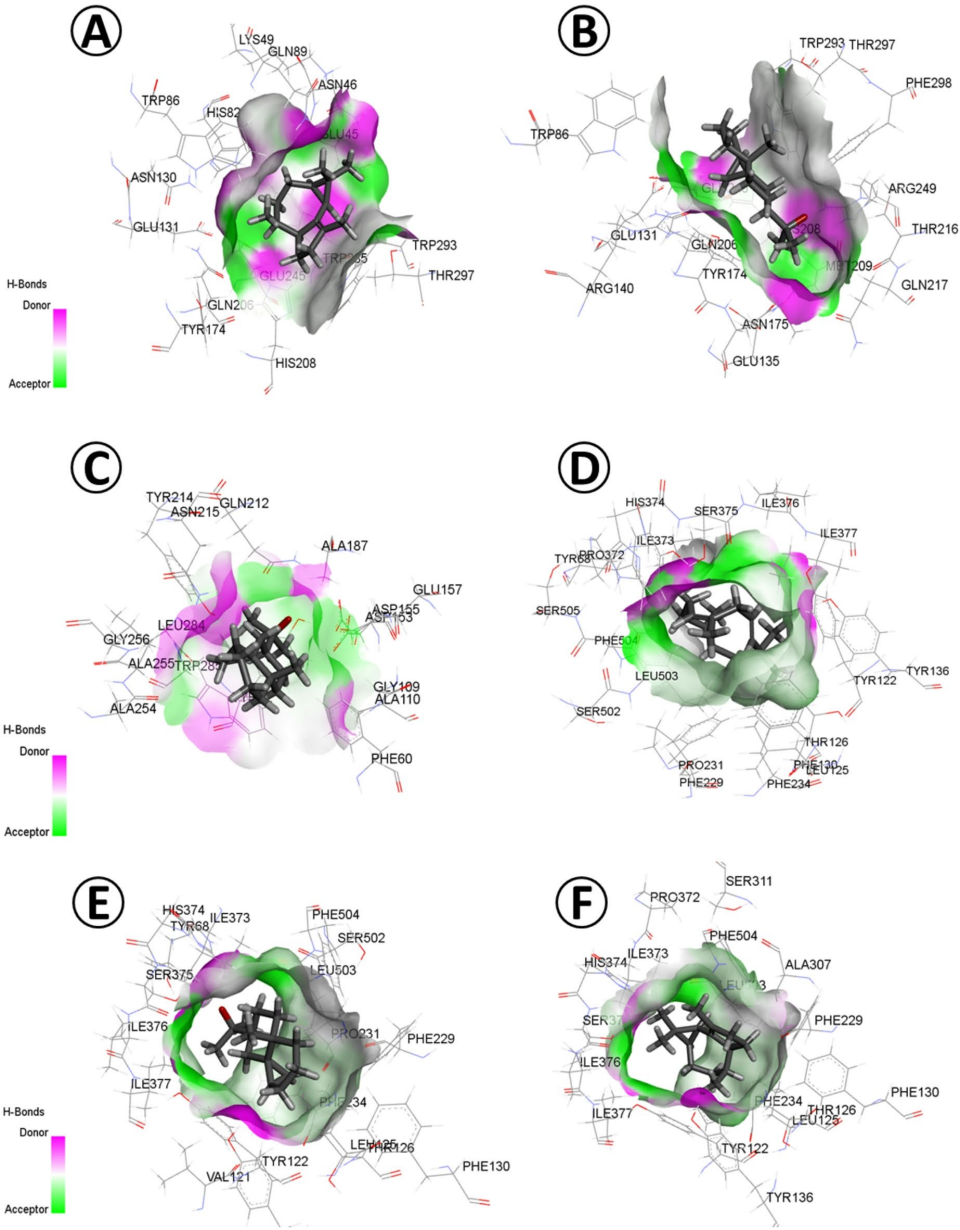
Molecular docking of *D. ismaelis Asch.* (**A**) 7.beta.-(1-hydroxy-1-methylethyl)-4a.beta.-methyl-1a. beta.-decahydro cyclopropa[d]naphthalene, and GH10 xylanase (PDB ID: 3U7B). (**B**) Alpha.-Gurjunene and GH10 xylanase. (**C**) 7.beta.-(1-hydroxy-1-methylethyl)-4a.beta.-methyl-1a. beta.-decahydro cyclopropa[d]naphthalene, and plant-type chitinase inhibitors (PDB ID: 4TXE). (**D**) Alpha.-Gurjunene and Plant-type chitinase inhibitors. (**E**) 7.beta.-(1-hydroxy-1-methylethyl)-4a.beta.-methyl-1a.beta.-decahydro cyclopropa[d]naphthalene and Sterol 14-alpha Demethylase (PDB ID: 5FRB). (**F**) Alpha.-Gurjunene and Sterol 14-alpha Demethylase.

*D. ismaelis* Asch. had a total of 40 phytochemicals, of which five constituents showed remarkable docking features against the three targeted enzymes (Fig. 7). Similarly, the screened phytochemicals, including 7. beta.-(1-hydroxy-1-methylethyl)-4a.beta.-methyl-1a. beta.-decahydro cyclopropa[d]naphthalene, 2-Naphthalenemethanol, 1,2,3,4,4a,5,6,7-octahydro-.alpha.,.alpha.,4a,8-tetramethyl-, (2R-cis)-, alpha-urjunene, azulene, 1,2,3,5,6,7,8,8a-octahydro-1,4-dimethyl-7-(1-methylethenyl)-, [1 S-(1. alpha.,7.alpha.,8a.beta.)] and Alpha.-Humulene revealed the highest values of binding free energy (kcal/mol) and inhibitory constant (µM) against GH10 xylanase with − 7.5 and 5.5, -7.3 and 5.35, -7.1 and 5.21, -7 and 5.13 and − 6.9 and 5.06, respectively. Relatively high ligand efficiency (kcal/mol/non-H atom) was detected for these interactions, reaching 0.473, while low torsional energy, especially with Alpha.-Gurjunene and Alpha.-Humulene ligands where 0.00 torsional energy was reported (Supplementary Table S3).

Screening of phytochemicals in *D. ismaelis* ethanomethanolic extracts showed relatively less affinity toward plant-type chitinase inhibitors. The highest binding free energy (kcal/mol) was 7. beta.-(1-hydroxy-1-methylethyl)-4a.beta.-methyl-1a. -decahydro cyclopropa[d] naphthalene (-6.4) followed, 2,6-Octadiene, 2,6-dimethyl (-6.2),(5E)-5-OCTADECENE (-6.2), linalool (-6.1) and 2,6-Octadiene, 1-(1-ethoxyethoxy)-3,7-dimethyl (-6.1). At the same time, high inhibitory constant (pKi) was obtained between the phytochemicals ligands and the targeted fungal enzymes (4.69, 4.55 and 4.47 µM, respectively). In contrast, moderate ligand efficiency (kcal/mol/non-H atom) and torsional energy values were reported.

For Sterol 14-alpha demethylase, the highest binding free energy (kcal/mol) was exposed to five phytoconstituents of *D. ismaelis*, including alpha-gurjunene, alpha-humulene, 7. beta.-(1-hydroxy-1-methylethyl)-4a.beta.-methyl-1a. beta.-decahydro cyclopropa[d]naphthalene, 2-Naphthalenemethanol, 1,2,3,4,4a,5,6,7-octahydro .alpha.,.alpha.,4a,8-tetramethyl-, (2R-cis) and 8-ISOPROPYL-1-METHYL-5-METHYLENE-1,6-CYCLODECADIENE (− 8.7, − 8.5, − 8.5, − 8.3 and − 8.2 kcal/mol, respectively). Similarly, a high inhibitory constant (pKi) was recorded for the top five phytoconstituents against sterol 14-alpha demethylase, which ranged between 6.38 and 6.01 µM. These values are consistent with the high ligand efficiency (kcal/mol/non-H atom) values, ranging between 0.580 and 0.5188. A notable torsional energy of 0.00 was obtained between Alpha.-Gurjunene and Alpha.-Humulene and Sterol 14-alpha demethylase (Supplementary Table S3).

### Molecular dynamics (MD)

#### Molecular dynamics (MD) between selected phytoconstituents of *E. vesicaria* L. cav. and GH10 xylanase (PDB ID: 3U7B)

**Fig. 8:**
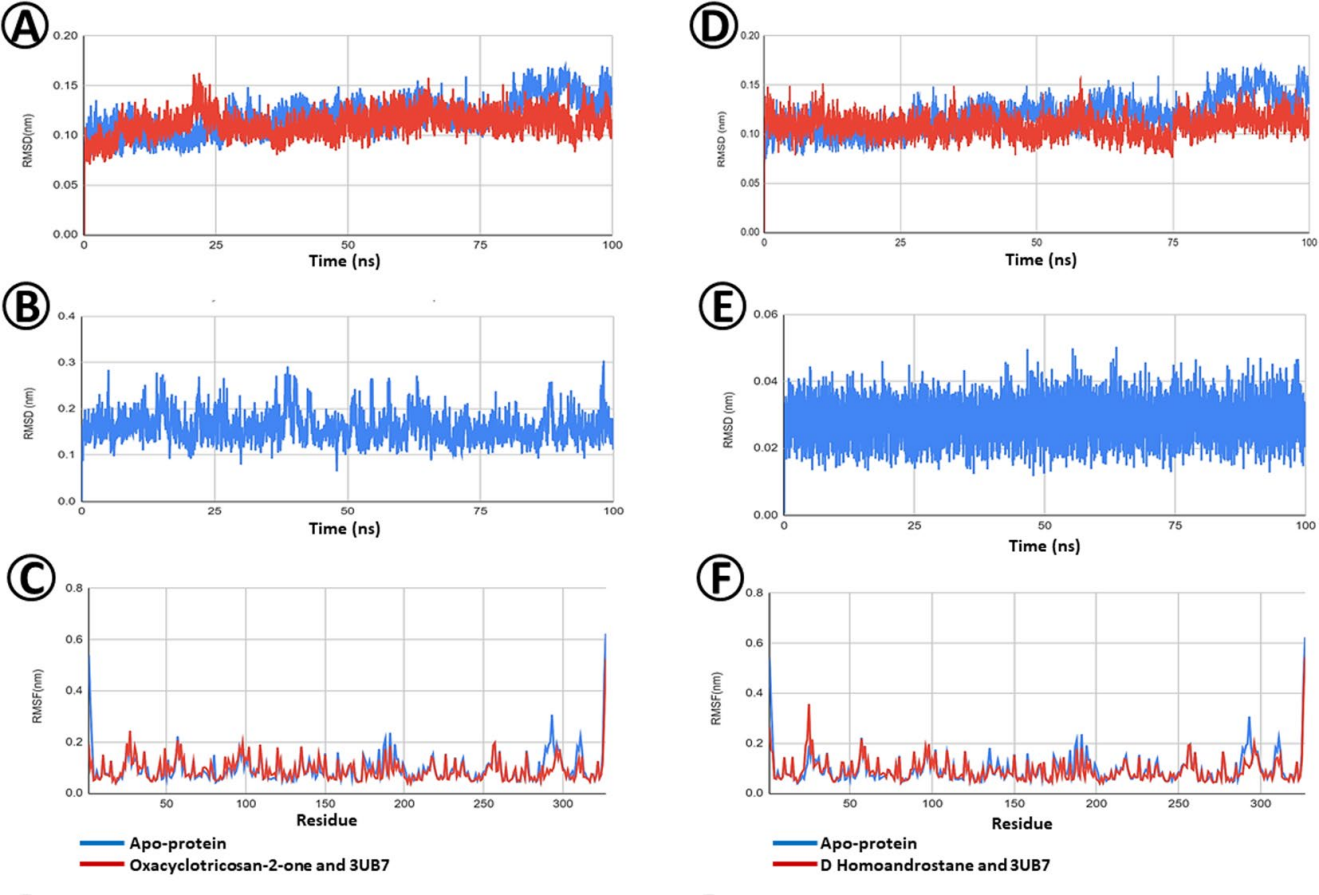
Molecular dynamics (MD) between selected phytoconstituents of *E. vesicaria* L. Cav. and GH10 xylanase (PDB ID: 3U7B) fungal protein. (**A**) Protein Root Mean Square Deviation (RMSD) of Oxacyclotricosan-2-one and GH10 xylanases. (**B**) Ligand Root Mean Square Deviation (RMSD) of D-Homoandrostane, (5.alpha.,13.alpha.) and GH10 xylanase. (**C**) Root Mean Square Fluctuation (RMSF) of Oxacyclotricosan-2-one and GH10 xylanase. (**D**) Protein Root Mean Square Deviation (RMSD) of D Homoandrostane, (5.alpha.,13.alpha.) and GH10 xylanase. (**E**) Ligand Root Mean Square Deviation (RMSD) of Oxacyclotricosan-2-one and GH10 xylanase. (**F**) Root Mean Square Fluctuation (RMSF) of D Homoandrostane, (5.alpha.,13.alpha.) and GH10 xylanase.

The Root mean square deviation (RMSD) (Fig. 8A) revealed the dynamic behavior of Oxacyclotricosan-2-one, showcasing fluctuations in the range of 0.2–0.3 nm which is generally considered to be within the acceptable range. Upon forming a complex with Oxacyclotricosan-2-one, it initially showed a modest increase in RMSD, potentially indicating an initial adjustment Maintaining an RMSD within the range of 0.15–0.2 nm (Fig. 8B).

Root Mean Square Fluctuation (RMSF) analysis (Fig. 8C) provided insights into the residue-specific dynamics of the entire simulation. Residues in the range–270–300 show slight fluctuations in the apoprotein, whereas the complex effectively stabilized these residues. This residue-specific impact of Oxacyclotricosan-2-one binding reduces the flexibility and contributes to the overall stability of the complex.

The Root mean square deviation (RMSD) of the protein in both bound and unbound state of the ligand D-Homoandrostane is presented in Fig. 8D. The GH10 xylanase apoprotein (PDB ID: 37UB) demonstrated remarkable stability throughout the simulation, maintaining an RMSD within the range of 0.15–0.2 nm. Root Mean Square Deviation (RMSD) analysis revealed the dynamic behavior of the ligand, indicating substantial fluctuations in the range of 0.4–0.5 nm (Fig. 8E).

Root Mean Square Fluctuation (RMSF) analysis clarified the residue-specific dynamics of the entire simulation (Fig. 8F). Residues in the range–280–300 show slight fluctuations in the apoprotein, whereas the complex effectively stabilized these residues. This residue-specific impact of D-Homoandrostane binding reduces flexibility and contributes to the overall stability of the complex.

#### Molecular dynamics (MD) between selected phytoconstituents of *E. vesicaria* L. cav. and plant-type chitinase inhibitors (PDB ID: 4TXE)

**Fig. 9:**
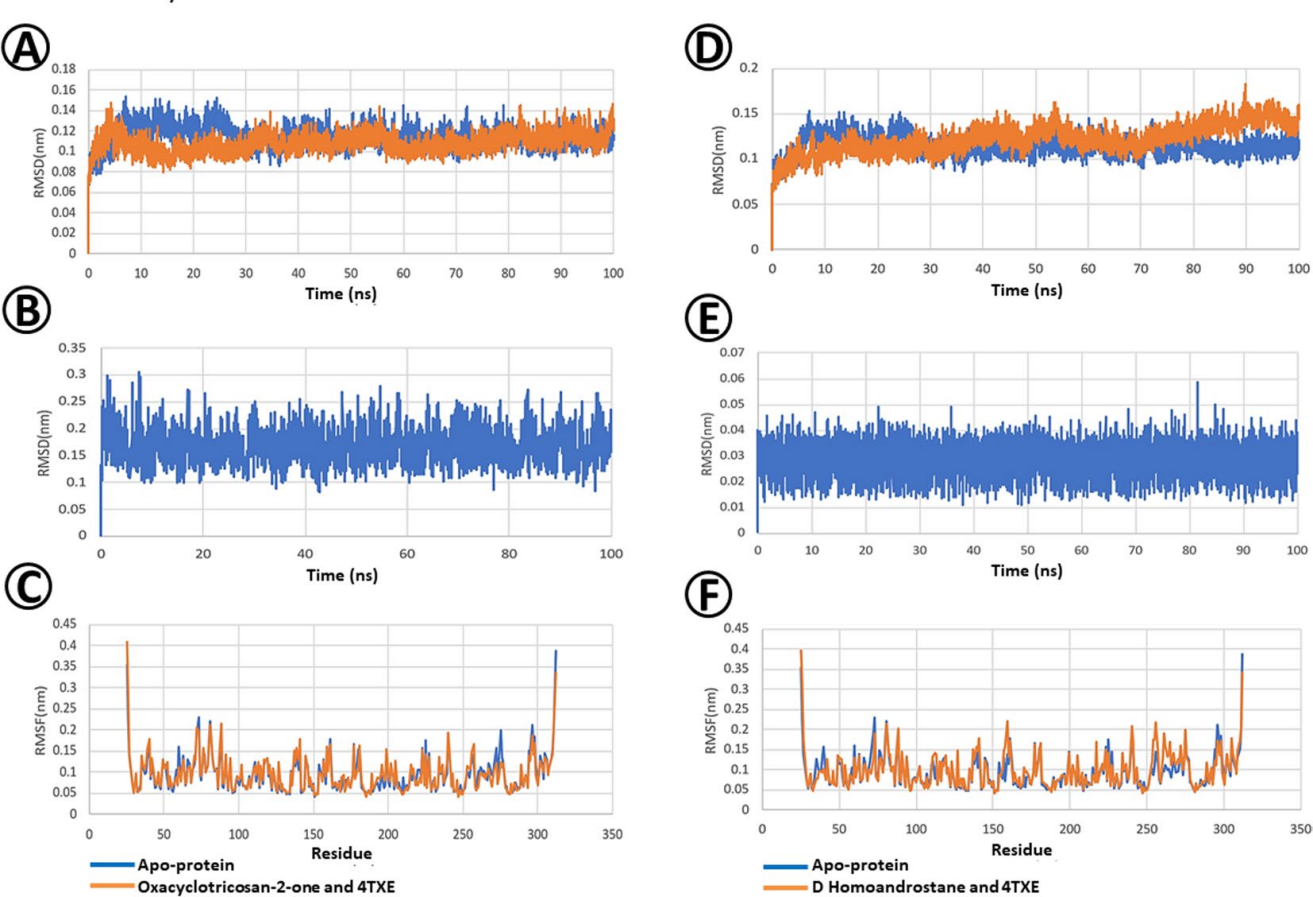
Molecular dynamics (MD) between selected phytoconstituents of *E. vesicaria* L. Cav. and Plant-type chitinase inhibitors (PDB ID: 4TXE) fungal protein. (**A**) Protein root-mean-square deviation (RMSD) of Oxacyclotricosan-2-one and Plant-type chitinase inhibitors. (**B**) Ligand Root Mean Square Deviation (RMSD) of D-Homoandrostane, (5.alpha.,13.alpha.) and Plant-type chitinase inhibitors. (**C**) Root Mean Square Fluctuation (RMSF) of Oxacyclotricosan-2-one and Plant-type chitinase inhibitors. (**D**) Protein Root Mean Square Deviation (RMSD) of D Homoandrostane, (5.alpha.,13.alpha.) and Plant-type chitinase inhibitors. (**E**) Ligand Root Mean Square Deviation (RMSD) of Oxacyclotricosan-2-one and Plant-type chitinase inhibitors. (**F**) Root Mean Square Fluctuation (RMSF) of D Homoandrostane, (5.alpha.,13.alpha.) and Plant-type chitinase inhibitors.

Root Mean Square Deviation (RMSD) comparison of protein (PDB ID: 4TXE) with ligand Oxacyclotricosan-2-one is depicted in Fig. 9A. The graph indicates that the protein-ligand complex remained highly stable, with RMSD values consistently falling within the acceptable range of 0.1–0.15 nm.

The Ligand RMSD graph depicting ligand Oxacyclotricosan-2-one in the target-bound Plant-type chitinase inhibitors fungal protein form across a dynamic 100ns environment reveals deviation values ranging from 0.1 to 0.3 nm (Fig. 9B). While deviations exceeding 0.3 nm are typically considered high for a ligand’s displacement from its initial pose, the consistent pattern observed here suggests a level of stability.

Residue-specific dynamics of the plant-type chitinase inhibitor fungal protein in both the Oxacyclotricosan-2-one bound and unbound forms are depicted in Fig. 9C with the representation of Root Mean Square Fluctuation (RMSF) analysis. Here, most of the residues are highly comparable, and a few residues that have slightly higher fluctuations (at 61 and 271st residues of the apoprotein) are stabilized by the ligand in the bound form.

Root Mean Square Deviation (RMSD) analysis was used to compare the behavior of proteins with and without D-homoandrostane (5. alpha.,13.alpha.) ligand over a 100 ns simulation (Fig. 9D). The graph indicates that the protein backbone deviates by less than 0.3 nm, a generally acceptable range in molecular dynamic simulations.

The Root Mean Square Deviation (RMSD) analysis of D-Homoandrostane, (5.alpha.,13.alpha.), in relation to its original conformation, demonstrates deviations are within the 0.05 nm which is the indication of a potential binder (Fig. 9E).

Figure 9Fshows the Root Mean Square Fluctuation (RMSF) analysis and sheds light on the residue-specific fluctuations of the entire simulation. The residue-specific impacts of d-homoandrostane (5. alpha.,13.alpha.) binding reduced flexibility and contributed to the overall stability of the complex at the end of the simulation. The RMSF findings complement the RMSD results, offering an understanding of how ligands influence distinct regions of the protein structure. Based on these observations, D-Homoandrostane, (5.alpha.,13.alpha.) is a promising binder for the target protein (Plant-type chitinase inhibitors), demonstrating comparable stability to the apoprotein.

#### Molecular dynamics (MD) between selected phytoconstituents of *E. vesicaria* L. cav. and sterol 14-alpha demethylase (PDB ID: 5FRB)

**Fig. 10:**
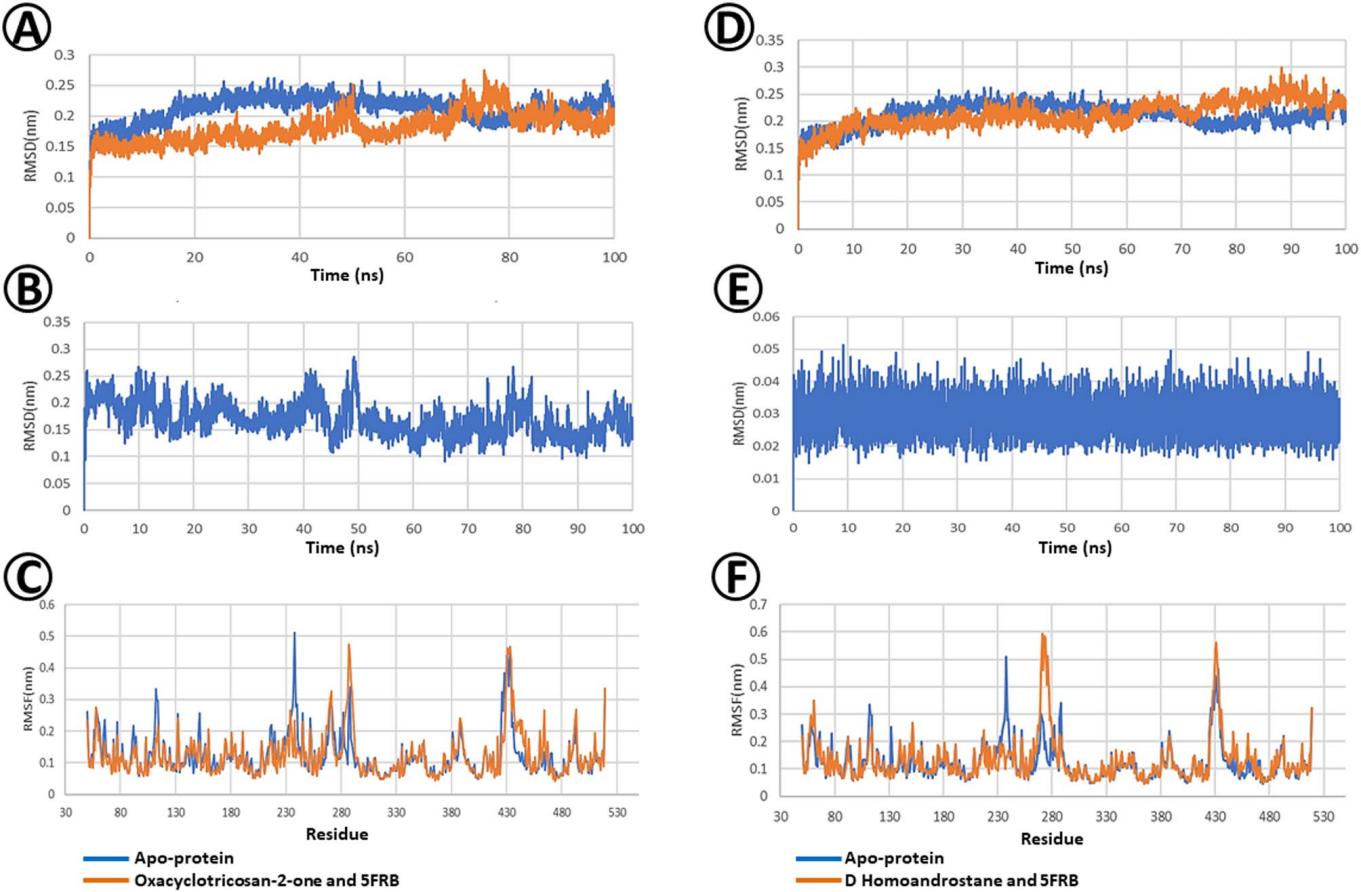
Molecular dynamics (MD) between selected phytoconstituents of *E. vesicaria* L. Cav. and Sterol 14-alpha Demethylase (PDB ID: 5FRB) fungal protein. (**A**) Protein Root Mean Square Deviation (RMSD) of Oxacyclotricosan-2-one and Sterol 14-alpha Demethylase. (**B**) Ligand Root Mean Square Deviation (RMSD) of D-Homoandrostane, (5.alpha.,13.alpha.) and Sterol 14-alpha Demethylase. (**C**) Root Mean Square Fluctuation (RMSF) of Oxacyclotricosan-2-one and Sterol 14-alpha Demethylase. (**D**) Protein Root Mean Square Deviation (RMSD) of D Homoandrostane, (5.alpha.,13.alpha.) and Sterol 14-alpha Demethylase. (**E**) Ligand Root Mean Square Deviation (RMSD) of Oxacyclotricosan-2-one and Sterol 14-alpha Demethylase. (**F**) Root Mean Square Fluctuation (RMSF) of D Homoandrostane, (5.alpha.,13.alpha.) and Sterol 14-alpha Demethylase.

Figure 10A depicts a comparison of Root Mean Square Deviation (RMSD) analysis for the Sterol 14-alpha Demethylase protein (PDB ID: 5FRB) in both bound and unbound conditions with the ligand Oxacyclotricosan-2-one over 100 ns simulation. The graph illustrates the deviation of the protein backbone below 0.3 nm, which is considered acceptable in a typical molecular dynamic simulation. Oxacyclotricosan-2-one stabilized the protein in most frames, except for those exhibiting an elevated RMSD between 75 and 80 ns.

Root Mean Square Deviation (RMSD) analysis revealed the dynamic behavior of Oxacyclotricosan-2-one (Fig. 10B), indicating substantial deviations in the range of 0.25–0.3 nm before 50 ns. Additionally, the deviations decreased to 0.15 nm which is evident for the efficient binding of Oxacyclotricosan-2-one to the target protein (Sterol 14-alpha demethylase).

Root Mean Square Fluctuation (RMSF) analysis (Fig. 10C) elucidated the residue-specific dynamics of the entire simulation. Residues in the range–230–250 show slight fluctuations in the apoprotein, whereas the complex effectively stabilized these residues.

In Fig. 10D, the Root Mean Square Deviation (RMSD) analysis compares the behavior of protein 5FRB with and without the D-Homoandrostane ligand over a 100 ns simulation. The graph indicates that the protein’s backbone deviates by less than 0.3 nm, a generally acceptable range in molecular dynamic simulations. Although D-homoandrostane effectively stabilized the protein in most frames, there were notable instances of elevated RMSD between 75 and 90 ns.

The Root Mean Square Deviation (RMSD) analysis of D-homoandrostane (Fig. 10E), in relation to its original conformation, demonstrated deviations within 0.05 nm which is an indication of a potential binder.

Figure 10F illustrates the Root Mean Square Fluctuation (RMSF) analysis, providing insights into residue-specific fluctuations of the entire simulation. The complex residues at 270–280 have shown high fluctuations but stabilized in the later stages. The residue-specific impact of d-homoandrostane binding reduced flexibility and contributed to the overall stability of the complex at the end of the simulation.

## Discussion

Fusarium is the most destructive fungal pathogen in maize and other major crops. Phenotypic analysis and molecular identification revealed that the isolated damping-off associated fungi belonged to different species within the Fusarium genus; however, *F. verticillioides* was more frequent than the other species. The pathogenicity assessment under in vitro conditions clearly showed that all tested isolates were pathogenic to sweet corn (Fig. 1). However, *F. verticillioides* (Fv-A), *F. fujikuroi* (Ff-A), and *F. oxysporum* (Fo-W2) isolates were more aggressive than the other isolates, revealing the highest significant rate (*P* ≤ 0.05) of pathogenicity against sweet corn seeds. The *in planta* assessment of pathogenicity (Fig. 2) showed significant differences between the examined fungal isolates with respect to germination (%), infection (%), and infection severity (%). These results were in line with the previous *in planta* assessment, in which *F. verticillioides*, *F. fujikuroi*, and *F. oxysporum* isolates had a higher negative effect on seed germination. These results clearly indicate that the three Fusarium species represent a serious threat to seed germination in sweet corn. Although a high infection rate (%) was observed for all the examined isolates, *F. verticillioides*, *F. oxysporum*, and *F. acuminatum* achieved a complete infection rate (100%). In contrast, *F. verticillioides* (Fv-A), *F. fujikuroi* (Ff-A), and *F. oxysporum* (Fo-W2) showed notable aggressiveness, with 91.67%, 90%, and 86.67% infection severity in sweet corn seedlings, respectively.

The germination/emergence stage found to be the most vulnerable stage compared to less-affected older stages of plant life. *F. verticillioides* is mainly transmitted via contaminated seeds and can cause serious damage to maize crop during the pre- or post-emergence stages. The symptomless systemic infection with *F. verticillioides* can be initiated under a broad range of environmental conditions; however, this phenomenon has varied greatly based on the individual study^18^. The authors confirmed that *F. verticillioides* caused significant damage to *Pinus* spp. in Northwest Spain. However, Pajares and Díez^19^ reported that *F. verticillioides* had damping-off damage similar to that induced by *F. oxysporum*. The first report of *F. verticillioides* being associated with damping-off disease in corn was documented in Taiwan by Sun et al.^20^. These reports indicate that weak and stunned growth ended with the death of infected seedlings.

The antagonistic assessment of Arugula (*E. vesicaria* L. Cav.), White goosefoot (*C. album* L.), African mustard (*S. africana* L. Botsch.), Bermuda buttercup (*O. pes-caprae* L.), and Ducrosia (*D. ismaelis* Asch.) was tested at three concentrations (1 mg mL^− 1^, 2 mg mL^− 1^ and 3 mg mL^− 1^) against three selected fungal isolates (*F. verticillioides* (Fv-A), *F. fujikuroi* (Ff-A), and *F. oxysporum* (Fo-W2), (Fig. 3). The results revealed that *F. verticillioides* (Fv-A) was more aggressive by recording the highest pathogenicity under in vitro and *in planta* conditions. The pathogenicity of most fungi is highly depending on the amount and the type of secreted enzyme. Moreover, inoculum potential of seed-born fungal pathogens could effectively determine their ability to negatively affect seed emergence^21^ especially *F. verticillioides* which showed higher aggressiveness at early stages of plant life by causing radical decay^22^.

Regarding the ethanomethanolic extract, the obtained results indicated a distinctive performance for *E. vesicaria*, *D. ismaelis*, and *O. pes-caprae* in controlling the three selected fungal isolates under in vitro conditions compared to negative and positive controls. This exceptional attitude was more pronounced at the highest concentration (3 mg mL^− 1^), which differed significantly from that of the other concentration treatments (Fig. 3). These results may be attributed to the large number and diversity of phytoconstituents present in these extracts, especially those known for their antimicrobial and/or antioxidant activities. In the case of synthetic pure compounds, the greatest determinant of their antimicrobial activity is the presence of a specific antagonistic pathway. Comparatively, plant extracts, especially those derived from wildly growing types can expose different mechanism of action against fungi and other microorganisms. The combined impact of phytoconstituents may contribute to synergistic interactions with strong bioactivity even at relatively low concentrations^23^.

The *in planta* antagonistic assessment of the selected plant extracts (*E. vesicaria* L. Cav., *O. pes-caprae* L. and *D. ismaelis* Asch.) were consistent with those obtained from in vitro investigations. In general, *F. verticillioides* (Fv-A) was more aggressive than the other two fungal isolates, negatively affecting almost all the studied growth variables of sweet corn (germination (%), infection (%), infection severity (%), plant height (cm), fresh weight (g), dry weight (g), and chlorophyll content). On the other hand, plant extracts that showed significant positive effect in enhancing growth variables especially *E. vesicaria* L. Cav. showed superiority over the other plant extracts.

In the current investigation, phytochemical analysis using GC-MS indicated that the five plant extracts had different phytochemical profiles. The different chemical compositions were mainly attributed to the different plant types and families to which they belong. The wide range of detected phytoconstituents can be categorized as fatty acids, alkanes, esters, terpenoids, flavonoids, glycosides, sterols, phenolics, saponins, and heterocyclic compounds. According to the percentage of area (%), the most abundant compounds are marked with antimicrobial and/or antioxidant activity, especially 1-eicosanol^24^ heptacosane^25^ docosanoic acid, ethyl ester^26^ and Tetracosanol-1 from *E. vesicaria*^27^.

Some of the bioactive compounds identified from the ethanolic extract of *D. ismaelis* Asch. are known for their biological activity, especially those that exist in large quantities, such as nonadecane^28^ heneicosane^29^ and 8-ISOPROPYL-1-METHYL-5-METHYLENE-1,6-CYCLODECADIENE^30^. The chemical profile of *O. pes-caprae* L. includes diverse components that have recently been found to have antimicrobial characteristics, such as Z)6,(Z)9-Pentadecadien-1-ol^31^ n-hexadecanoic acid^32^ and celidoniol and deoxy^33^.

Through molecular docking investigation, the binding free energy (kcal/mol), inhibition constant pKi (µM), ligand efficiency (kcal/mol/non-H atom) and torsional energy was estimated between the phytoconstituents (ligand) from *E. vesicaria* L. Cav., *Ox. pes-caprae* L. and *D. ismaelis* Asch. and three fungal enzymes (target), namely GH10 xylanase (PDB ID: 3U7B), plant-type chitinase inhibitors (PDB ID: 4TXE), and Sterol 14-alpha demethylase (PDB ID: 5FRB), (Supplementary Table S3).

Oxacyclotricosan-2-one and D-Homoandrostane from *E. vesicaria* L. Cav. The ethanomethanolic extract showed the best docking results, where high binding free energy, relatively low inhibition constant (pKi), ligand efficiency, and zero torsional energy against the three targeted fungal enzymes were detected. The distinctive molecular docking parameters revealed by Oxacyclotricosan-2-one and D-Homoandrostane indicate their exceptional ability to bind to and thus effectively inhibit fungal infection-related enzymes. Therefore, these bioactive compounds offer novel and safe sustainable alternatives for the design of new fungicides to manage plant disease caused by phytopathogenic fungi.

A wide range of docking parameters was revealed by *O. pes-caprae* L. phytoconstituents, of which the binding free energy reached the highest values between the three ligand proteins and each of Vitamin E and Benzenepropanoic acid, 3,5-bis(1,1-dimethylethyl)-4-hydroxy-, methyl ester. Similarly, these ligands had the highest pKi values, while moderately low ligand efficiencies and torsional energies were observed (Supplementary Table S3).

Remarkable molecular dynamics parameters have been detected for some phytoconstituents of *D. ismaelis* and three target enzymes. The screened phytochemicals including 7.beta.-(1-hydroxy-1-methylethyl), Alpha.-Gurjunene in addition to Alpha.-Humulene (Supplementary Table S3). These ligands exhibited the highest binding free energy, whereas relatively low values were observed for the inhibition constant (pKi), ligand efficiency, and torsional energy.

Regarding kinetics and dynamics properties, the Root Mean Square Deviation (RMSD) below 0.3 nm is considered acceptable for measuring the protein’s deviation from its original position during dynamic simulations. In this case, the protein-bound and unbound forms are highly comparable, and the bound form of the protein has remarkably stabilized the protein even better than the apoprotein in the initial simulation time and continues to do so over the entire dynamics. Overall, the consistent stability of the complex and its convergence with the apoprotein support the conclusion that the selected *E. vesicaria* L. Cav. Oxacyclotricosan-2-one and D-homoandrostane ligands effectively interact with and influence the behavior of GH10 xylanase (PDB ID: 3U7B), (Fig. 8A, D), plant-type chitinase inhibitors (PDB ID: 4TXE) (Fig. 9A, D), and sterol 14-alpha demethylase protein (PDB ID: 5FRB), (Fig. 10A, D). Thus, Oxacyclotricosan-2-one and D-Homoandrostane are potential binders of these target proteins.

Upon forming a complex with d-homoandrostane, the target protein showed similar stability. However, the complex stabilized over time, consistently exhibiting lower RMSD values than the apoprotein. This suggests a stabilizing effect resulting from the binding interaction, highlighting the potential efficacy of D-Homoandrostane as a good binder of the target proteins (Fig. 8D). D-Homoandrostane is not only a good binder, but also an excellent one, showing a remarkable affinity for the sterol 14-alpha demethylase fungal protein.

The RMSF findings (Figs. 8, 9 and 10) complement the RMSD results, offering a reasonable idea of how the ligand influences the distinct regions of the protein structure. The observed consistency of Oxacyclotricosan-2-one and D-Homoandrostane ligands in the target bound of the selected fungal proteins, particularly plant-type chitinase inhibitors, may be attributed to significant pose corrections; notably, the ligand remains within the binding pocket throughout the entire simulation. Despite the values approaching the higher threshold, the sustained presence of the ligand in the binding site indicated its potential as a strong binder, emphasizing the reliability of the interaction in the simulated environment.

## Materials and methods

### Sampling of infected sweet corn plants

A total of sweet corn seedlings showing discoloration, browning, necrosis, and wilting symptoms (Supplementary Fig. S7) were collected from three distinct governorates through the country, namely Anbar (33° 25’ 42.9” N 43° 20’ 13.6” E), Tikrit (34.5990°"N, 43.6750°"E), and Wasit (33° 00’ 45.3” N, 44° 49’ 24.6” E) during March and April 2023 (Supplementary Fig. S8). The collected plant samples were preserved in polyethylene bags marked with all the necessary details and brought directly to the laboratory to isolate companion pathogenic fungi.

### Isolation and phenotypic identification of pathogenic fungi

The collected sweet corn seedlings were washed carefully with running tap water to remove the attached dirt and fine impurities. Cleaned stem bases and roots were sliced into small fragments (0.5–1 cm) and then immersed in sodium hypochlorite NaOCl (1% free chlorine) at a concentration of 5% for surface disinfection. After 3 min, the fragments were thoroughly rinsed with sterile water for 2 min and left on aseptic filter paper until completely dried.

The dried plant fragments were cultured on Potato Dextrose Agar (PDA) medium amended with antibiotic (Amoxicillin 200 mg L^− 1^), and autoclaved at (121 °C 1.5 kg cm^34^) for 20 min. Plates were incubated at 25 ± 1 °C for 7 days and checked daily. The purification was conducted under aseptic conditions by carefully transferring the growing hyphae tips from each fungal colony to new PDA plates using a sterile needle. The cultured plates were incubated for five days at 25 ± 1 °C for subsequent phenotypic identification based on fungal culture, mycelium, macroconidia, and microconidia to morphologically distinguish between the isolated pathogenic fungi.

### Molecular identification of pathogenic fungi

The fungal genomic DNA was extracted from pure fungal colony using ZR Fungal/Bacterial DNA MiniPrep™ (Zymo Research, Irvine, CA 92614, USA) extraction kit. The manufacturer’s instructions were followed literally. The quality and quantity of the extracted DNA were checked by estimating the absorption ratio using a NanoDrop 2000 spectrophotometer (Thermo Scientific, Wilmington, NC, USA) according to the following formula:

$$\:DNA\:purity\:ratio=O.D.260/O.D.280$$

Molecular identification of the associated pathogenic fungi was performed using polymerase chain reaction (PCR) with the aid of two sets of oligonucleotides specifically designed to target the Internal Transcribed Spacer (ITS) of rDNA^35^ and Translational Elongation Factor 1-a (TEF1-a)^36^. The ITS1 (5′-TCCGTAGGTGAACCTGCGG-3′) and ITS4 (5′-TCCTCCGCTTATTGATATGC-3′) were used as forward and reverse primers to amplify the ITS region, while EF-F (5’-GTTAAGAGGCGCGGTGTCGGTGTG-3’) and EF-R (5’-GGAAGTACCAGTGATCAT GTT-3’) were used as forward and reverse primers to amplify the TEF1-a region (Integrated DNA Technologies, Inc., Coralville, Iowa, USA).

The PCR mixtures were performed in a 25 µl final reaction volume for each, including 5 µl of Taq PCR PreMix, and 1 µl of each of the forward and reverse primers (10 pmol) in addition to 1.5 µl of genomic DNA. Deionized distilled water (DDW) was added to complete the reaction volume. The T100 Thermal Cycler Bio-Rad (Hercules, California, USA) was used to amplify the targeted ITS conservative region under the following conditions: initial denaturation at 94 °C for 3 min, then 35 cycles of denaturation at 94 °C for 45s, followed by annealing at 52 °C for 1 min, 72 °C for 1 min, and final extension at 72 °C for 7 min. To amplify the TEF1-a region, the following thermal profile was applied: initial denaturation at 94 °C for 85 s, then 35 cycles of denaturation at 94 °C for 45 s, followed by annealing at 59 °C for 1 min and extension at 72 °C for 1 min, followed by final extension at 72 °C for 10 min. The PCR-amplified products were separated by agarose gel electrophoresis (1.5%) with a 1 kb ladder. After electrophoresis and red staining, ultraviolet light (302 nm) was used to visualize the separated DNA fragments.

The purified ITS and TEF1-a fragments were sequenced using 3730XL Genetic Analyzer-Applied Biosystems (Waltham, Massachusetts, USA), in the National Instrumentation Center for Environmental Management—NICEM (http://nicem.snu.ac.kr/main/?en_skin=index.html)/the Biotechnology lab following Sanger sequencing protocol^37^. The homology test was conducted using Basic Local Alignment Search Tool (BLAST) software at the National Center Biotechnology Information (NCBI) (http://www.ncbi.nlm.nih.gov) and Molecular Evolutionary Genetic Analysis (MEGA7) software.

### In vitro assessment of fungal pathogenicity

The pathogenicity of the 14 fungal isolates was assessed in sweet corn seeds grown on Potato Dextrose Agar (PDA). PDA medium was prepared at a rate of 20 g L^− 1^ and bacterial growth was inhibited by amoxicillin addition (200 mg L^− 1^), and then autoclaved at 121 °C, and 1.5 kg cm^34^ for 20 min. Next, PDA was poured into 9 cm plates, left to cool and solidify before inoculated with a 5 mm disc of pure fully grown fungal colony. Sweet corn seeds were superficially disinfected with 5% NaOCl (1% free chlorine) for 3 min, rinsed with sterile water for 2 min, left to dry on aseptic filter paper, and finally ten seeds were distributed on 9 cm plates circularly containing PDA medium. Each treatment was replicated three times, in addition to the control group, which had no pathogenic fungus. Treatments were assigned randomly to experimental units using a completely randomized design (CRD). According to the least significant differences (LSD), the mean differences were investigated at 0.05 level of probability (*P ≤ 0.05*) and all data were subjected to analysis of variance (ANOVA). All treatments were incubated at 25 ± 1 °C, and ten days later, the percentage of germinated seeds was estimated using the following formula:

$$\:Seed\:germination\:\left(\%\right)=\frac{No.\:\:of\:germinated\:seeds}{No.\:\:of\:total\:seeds}\times\:100$$

### Fungal inoculum preparation

The fungal inoculum was prepared from seven days old pure culture for each isolate. Local millet seeds were cleaned and rinsed repeatedly with tap water before being drenched for five hours. The excess water was discarded and cleaned millet seeds were evenly distributed in autoclavable flasks (250 mL volume) and autoclaved twice, each for 20 min (121 °C, 1.5 kg cm^34^). After cooling, the autoclaved flasks containing millet seeds were inoculated with discs (5 mm) of each pure fungal culture and incubated at 25 ± 1 °C for two weeks. The incubation step was interrupted by daily stirring to ensure fungal colonization of the millet seeds.

### In planta assessment of fungal pathogenicity

The pathogenicity of the isolated fungi was assessed in pots under field conditions. Initially, the sterilization solution was prepared by adding formalin to distilled water at a ratio of 1:50 (v/v), then added to wet the previously prepared soil: peat moss mixture 2:1 (w/w). Next, the soil was covered with a polyethylene sheet, closed tightly, and left covered for ten days under field conditions. The polyethylene sheet was then removed, and the soil was stirred well under sunlight for three days to ensure complete volatilization of the sterilization solution. Disinfected soil was distributed in 10 kg clean plastic pots. The inoculum of each pathogenic fungi was added to each plastic pot separately at a rate of 1% (w/w), then pots were adequately watered, tightly covered with polyethylene bags, and left for the next three days. Subsequently, sweet corn seeds were superficially sterilized with NaOCl (5%) for 3 min, rinsed with sterile water, naturally dried, and then sowed at a rate of 10 seeds per pot. The control treatments were prepared following the same previously detailed steps, but in the uncontaminated soil. Each treatment was repeated in triplicate and randomly distributed in a completely randomized design (CRD). Mean differences were tested using 0.05 LSD level of probability (*P ≤ 0.05*) and all data were subjected to analysis of variance (ANOVA). The seed germination percentage was calculated three times until complete germination of control treatment. Five weeks later, the percentage of infections was calculated using the following formula:

$$\:\text{P}\text{e}\text{r}\text{c}\text{e}\text{n}\text{t}\text{a}\text{g}\text{e}\:\text{o}\text{f}\:\text{i}\text{n}\text{f}\text{e}\text{c}\text{t}\text{i}\text{o}\text{n}\:\left(\text{\%}\right)\:=\frac{Number\:of\:infected\:plants}{Number\:of\:total\:plants}100$$

### Phytochemical analysis

#### Collection of plant materials

At blooming stage, the aerial parts of five native plant species namely, Arugula (*Eruca vesicaria* L. Cav.), African mustard (*Strigosella africana* L. Botsch.), White goosefoot (*Chenopodium album* L.), Bermuda buttercup (*Oxalis pes-caprae* L.), and Ducrosia (*Ducrosia ismaelis* Asch.) were collected in March 2023. The necessary approvals were obtained from the Environment Department in Anbar province to collect the plant samples that were packed in sealable plastic bags labeled with all necessary details (Supplementary Table S4). Specimens were identified by Prof. Dr. Ali Fadaam Almehemdi at the Center of Desert Studies, University of Anbar, Iraq. Voucher specimens of plants were deposited in the National Herbarium of Iraq with voucher numbers 60,971, 60,972, 60,973, 60,974 and 60,975 for *E. vesicaria* L., *S. africana* L., *C. album* L., *O. pes-caprae* L., and *D. ismaelis*, respectively. Plant materials were transferred directly to the postgraduate lab at the Department of Plant Protection, College of Agriculture, University of Anbar, for phytochemical analysis. After thorough washing with running tap water, the freshly collected plant samples were shade-dried at room temperature until a constant weight was reached. The dried plant material was grinded to a fine powder using an electric home grinder, sieved to remove undesired impurities, and preserved in dry airtight polyethylene bags at 4 °C until use.

### Preparation of plant ethanomethanolic extracts

Primarily, a total volume of 1 L of solvent mixture was prepared by equally 1:1 (v/v) adding 500 mL of absolute ethanol (99.9%) to 500 mL of absolute methanol (97.9%). The ground plant material was macerated at a ratio of 1:10 (w/v), where 100 g of each plant powder was refluxed separately in 1 L of ethanomethanolic solvent solution using a stirrer device for 10 min and left to rest overnight. The mixture was then placed in an Ultrasonic Bath Sonicator (50 kHz) at 35 °C for four hours. The resulting homogenate was vacuum-filtered twice using a Buchner funnel, first through a clean cloth and then through Whatman No.1 filter paper (Whatman, Kent, UK). The final filtrates (Supplementary Fig. S9) were completely dried with rotary evaporator equipment (Bibby Scientific Ltd., Stone ST15 0SA, UK) then weighted to determine the extracted mass from each plant species that was 14, 12, 16, 15 and 14 g for *E. vesicaria* L., *S. africana* L., *C. album* L., *O. pes-caprae* L. and *D. ismaelis*, respectively.

#### Gas chromatography–mass spectrometry analysis (GC-MS)

Gas chromatography-mass spectrometry analysis (GC-MS) was used to characterize the phytochemical profiles of the three selected plant extracts using GC-MS-QP2010 plus equipment (Shimadzu, Kyoto, Japan). The equipment was supplied with an autoinjector and a 5 MS capillary non-polar column of 30 mm × 0.25 mm inner dimension with 0.25 μm film thickness. The carrier gas was helium, adjusted to achieve 1.15 mL min^− 1^ flow rate of mobile phase. A 70 eV ionization system was used for mass spectroscopic analysis. The initial temperature was set at 80 °C for 2 min, then progressively increased to 280 °C for 5 min. Split mode was followed for sample injection at 250 °C. The isolated phytochemical constituents were characterized by comparing their mass spectra and retention indices with the National Institute of Standards and Technology (NIST14) and Wiley 10th/NIST 2014 mass spectral library (W10N14) reference mass spectral databases.

### In vitro assessment of plant extracts antagonistic effect

The antagonistic effect of the five plant extracts (*E. vesicaria* L., *S. africana* L., *C. album* L., *O. pes-caprae* L., and *D. ismaelis*) at three concentrations was assessed against three fungal isolates of *Fusarium* spp. (*F. fujikuroi*,* F. verticillioides*, and *F. oxysporum)* using inhibition tests following the disc diffusion method.

A laboratory experiment was carried out in late December 2023 in the postgraduate laboratory of the Department of Plant Protection, College of Agriculture, University of Anbar. Working solutions of 3 mg mL^− 1^ were prepared by dissolving 300 mg of each plant extract in 100 mL of 2.5% dimethyl sulfoxide (DMSO; Sigma-Aldrich Co., MO, USA) and diluted to obtain 1, 2, and 3 mg mL^− 1^ concentrations, respectively. The final dilutions were subjected to cold sterilization using Millipore filter paper (0.45 μm). Then, before solidification, two milliliters of each plant extract treatment were added to plates containing PDA nutrient medium, in addition to the negative control group where only DMSO was added. After solidifying, three plates were inoculated in the middle with a single 5 mm disc of actively growing culture of each fungal isolate; however, the positive control treatment was treated with azoxystrobin (12 µg). Finally, the inoculated plates were incubated at 25 ± 1 °C for 7 days and checked periodically. Radial mycelial growth (IR) was adopted to estimate the inhibitory effect of different treatments, in which the fungal colony diameter was recorded over 7 consecutive days, and the results were recorded based on the negative fungal colony control.

The growth inhibition percentage of the tested fungi was calculated as described by Pinto et al.^38^ as follows:

$$\:IR\:\left(\%\right)\:=\frac{Dc-Dt}{Dc}100$$

where Dc = diameter of the fungal colony in the negative control, Dt = diameter of the fungal colony in the different treatments.

Treatments of the five plant extracts at three concentrations were randomly allocated in factorial arrangement with complete randomized design (CRD) in three replicates for each. Mean significant differences between factorial treatments were checked using LSD at 0.05 level of probability (*P* ≤ 0.05). All data were subjected to analysis of variance (ANOVA).

### In planta assessment of plant extracts antagonistic effect

Based on the results of the in vitro assessment of the antagonistic effect of the five used plant extracts, three plant extracts namely, *E. vesicaria* L. Cav., *O. pes-caprae* L., and *D. ismaelis* Asch. were selected for further assessment of plant extracts antagonistic effect against three selected fungal isolates (*Fusarium fujikuroi* (Ff-A), *Fusarium verticillioides* (Fv-A), and *Fusarium oxysporum* (Fo-W2)). The *in planta* investigation was conducted in plastic pots under field conditions following the previously described steps in the *in planta* assessment of fungal pathogenicity in the same order and conditions. Plant extracts were prepared at a concentration of 3 mg mL^− 1^ according to the in vitro assessment of plant extracts. The plant extracts were applied after soil inoculation and before seed sowing at a rate of 1% (v/w). The negative and positive control groups included DMSO and azoxystrobin (12 µg), respectively. After 45 days, data were recorded for germination (%), infection (%), infection severity (%), plant height (cm), fresh weight (g), dry weight (g), and chlorophyll content.

The severity of infection was calculated according to the pathological index, scaled to 5 degrees: 0 = healthy roots with no symptoms, 1 = 1–25% root discoloration, 2 = 26–50% roots discoloration, 3 = 51–75% root discoloration, 4 = 76–100% roots discoloration or damping-off, and 5 = rotten seeds.

The severity of the infection was calculated according to the following index:

$$\:\text{D}\text{i}\text{s}\text{e}\text{a}\text{s}\text{e}\:\text{S}\text{e}\text{v}\text{e}\text{r}\text{i}\text{t}\text{y}\:\text{I}\text{n}\text{d}\text{e}\text{x}\:\left(\text{D}\text{S}\text{I}\right)=\frac{\left(Number\:of\:plants\:with\:degree\:00\right)\dots\:\left(Number\:of\:plants\:with\:degree\:55\right)}{Number\:of\:total\:plants5}100$$

Treatments of the studied factors were randomly allocated in factorial arrangement with complete randomized design (CRD) in three replicates for each. Mean significant differences between factorial treatments were checked using LSD at 0.05 level of probability (*P* ≤ 0.05). All data were subjected to analysis of variance (ANOVA).

### In silico methodology

#### Protein 3D structures preparation

Three target fungal enzymes (proteins) were selected from the Protein Data Bank (PDB) IDs 3U7B (GH10 xylanase), 4TXE (plant-type chitinase inhibitors), and 5FRB (sterol 14-alpha demethylase) for in silico studies. The missing atoms were resolved using the Protein Repair & Analysis Server (PRAS), (www.protein-science.com)^39^. Structures with missing residues or breakages were subjected to homology modeling, loop refinement using the MODELLER 9.25 software, and energy minimization methodology using the steepest descent algorithm. The protein structures were subjected to the protein preparation protocol of the AutoDock 4.2.6 tool to generate pdbqt files for molecular docking.

#### Ligands preparation

The natural phytochemicals in the three selected ethanomethanolic extracts were considered in this study. The 3D structures of the phytochemical compounds were generated by SMILES^40^ using the OpenEye tool (https://www.eyesopen.com/). The structures were further energy minimized to obtain the least energy (stable) conformer using Merck Molecular Force Field (MMFF94). Subsequently, the energy-minimized ligand structures were subjected to ligand preparation to generate pdbqt files.

#### Molecular docking

The ligands of the three selected plant extracts were docked to the targets by generating docking grid parameters (box) for the selected pockets. Docking validation was performed by assigning the whole protein for docking (blind docking). The top three ligands from each sample with the lowest binding energy, large clusters, and better interactions with the target protein were combined with azoxystrobin, which is widely used as a fungicide.

#### Molecular dynamics simulations

The selected top-hit ligands and the reference fungicide (azoxystrobin) were subjected to molecular dynamics simulations. The selected protein-ligand complexes and apo-proteins were subjected to the AMBER99SB-ILDN version of the Assisted Model Building and Energy Refinement (AMBER) force field and the TIP3P water model in the GROMACS software. The simulation systems were prepared using a 1 nm cubic box imitating the biological cell environment at 300 K and 1 bar pressure.

The prepared MD systems were energy-minimized using steepest descent and conjugate gradient algorithms with 50,000 steps. The minimized MD systems were subjected to NVT and NPT equilibration procedures using a v-rescale thermostat and a c-rescale barostat with a total number of 500,000 steps equal to a 1ns time scale.

Finally, the equilibrated MD systems were subjected to a molecular dynamics simulation procedure on a 100ns time scale with a v-rescale thermostat and Parrinello-Rahman barostat using an md integrator.

## Conclusion

In conclusion, the present findings indicate an increased incidence of Fusarium spp., especially *F. verticillioides*, in sweet corn fields. These species had a developed ability to infect sweet corn during the pre- and post-emergence stages, causing damping-off disease. The plant extracts used efficiently reduced fungal pathogenicity because several bioactive compounds showed unique docking and dynamic properties. Oxacyclotricosan-2-one, D-Homoandrostane, Vitamin E, Benzenepropanoic acid, 3,5-bis(1,1-dimethylethyl)-4-hydroxy-, methyl ester; 7. beta.-(1-hydroxy-1-methylethyl) and alpha-gurjunene are potential binders with fungal target proteins and could be used in designing new fungicides.

## Electronic supplementary material

Below is the link to the electronic supplementary material.

Supplementary Material 1

Supplementary Material 2

Supplementary Material 3

Supplementary Material 4

Supplementary Material 5

## Data Availability

All data supporting the findings of this study are available within the paper and its Supplementary Information. The fourteen ITS and three TEF1-a sequences of the obtained Fusarium isolates were deposited in the National Center for Biotechnology Information (NCBI) (http://www.ncbi.nlm.nih.gov) with the accession numbers LC807017, LC807018, LC807019, LC807021, LC807022, LC807023, LC807024, LC807025, LC807026, LC807027, LC807028, LC807029, LC807031, and LC807032 for ITS sequences; and PV471465, PV471466 and PV471467 for TEF1-a sequences, respectively.
