# Supplementary material for: Investigating the efficacy of bioactive compounds from selected plant extracts against Gibberella fujikuroi species complex associated with damping off disease in sweet corn
Source: Sci Rep. 2025 Jul 1;15:21712. doi: 10.1038/s41598-025-05979-x (PMC12218985; doi:10.1038/s41598-025-05979-x)
Supplement: Supplementary file 1 — Supplementary Material 1 [file 41598_2025_5979_MOESM1_ESM.pdf]

Investigating the Efficacy of Bioactive Compounds from Selected Plant Extracts Against *Gibberella fujikuroi* Species Complex Associated With Damping off Disease in Sweet Corn

Alyaa Abd Ali, Ayoob Obaid Alfalahi*, Aalaa Khudhair Hassan[2], Ahlam Khalofah, Eilyn Mena, Abdelfattah A. Dababat, Fouad Mokrini

*** Corresponding authors: Ayoob Obaid Alfalahi (ag.ayoob.obaid@uoanbar.edu.iq)**

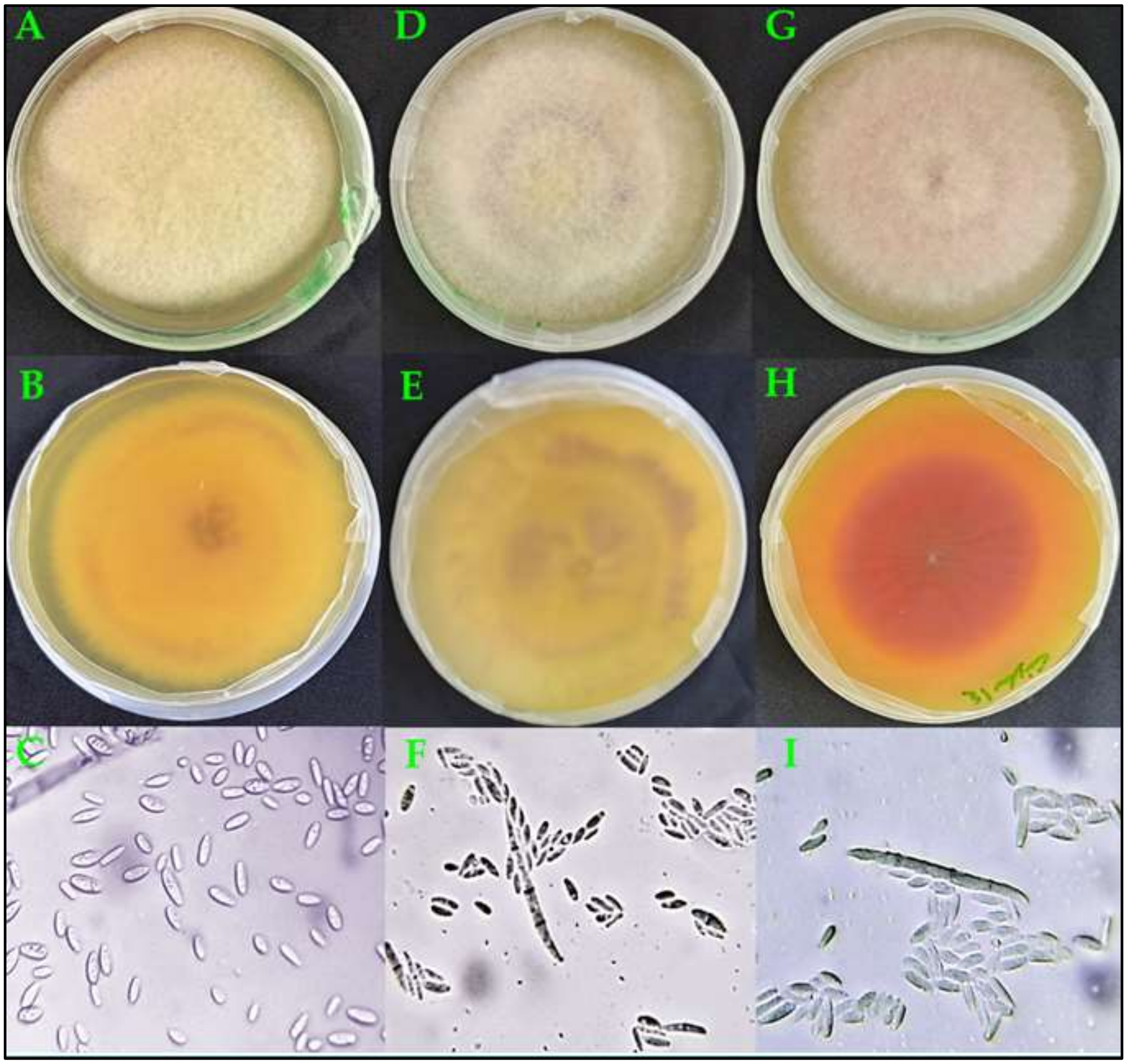

Figure S1: Morphological characterization of damping-off associated pathogenic fungi in sweet corn. A, B, and C: *Fusarium fujikuroi*. D, E, and F: *Fusarium oxysporum*. G, H, and I: *Fusarium verticillioides*.

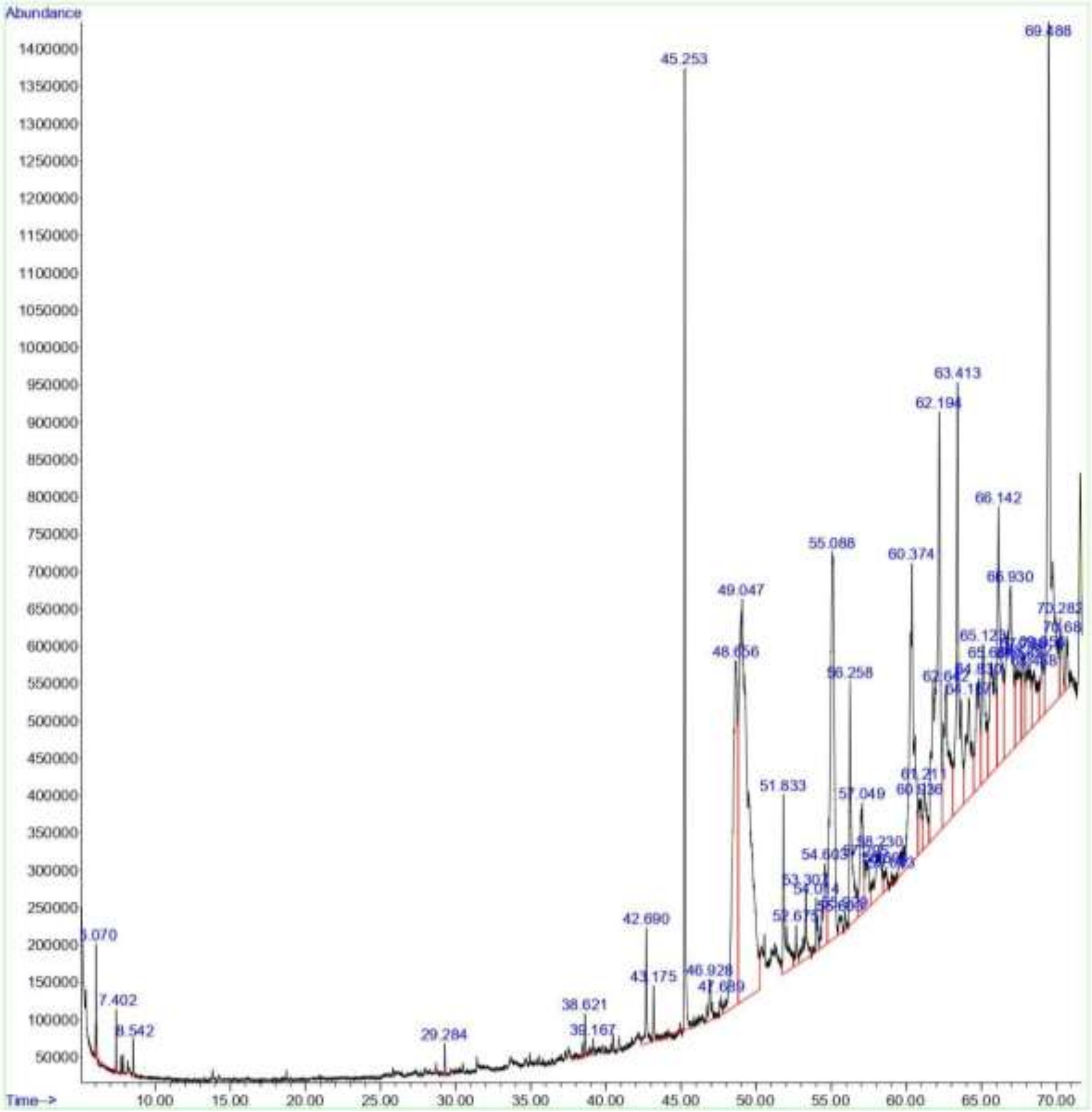

Figure S2: GC-MS profile of ethanomethanolic extract of *Eruca vesicaria* L. Cav.

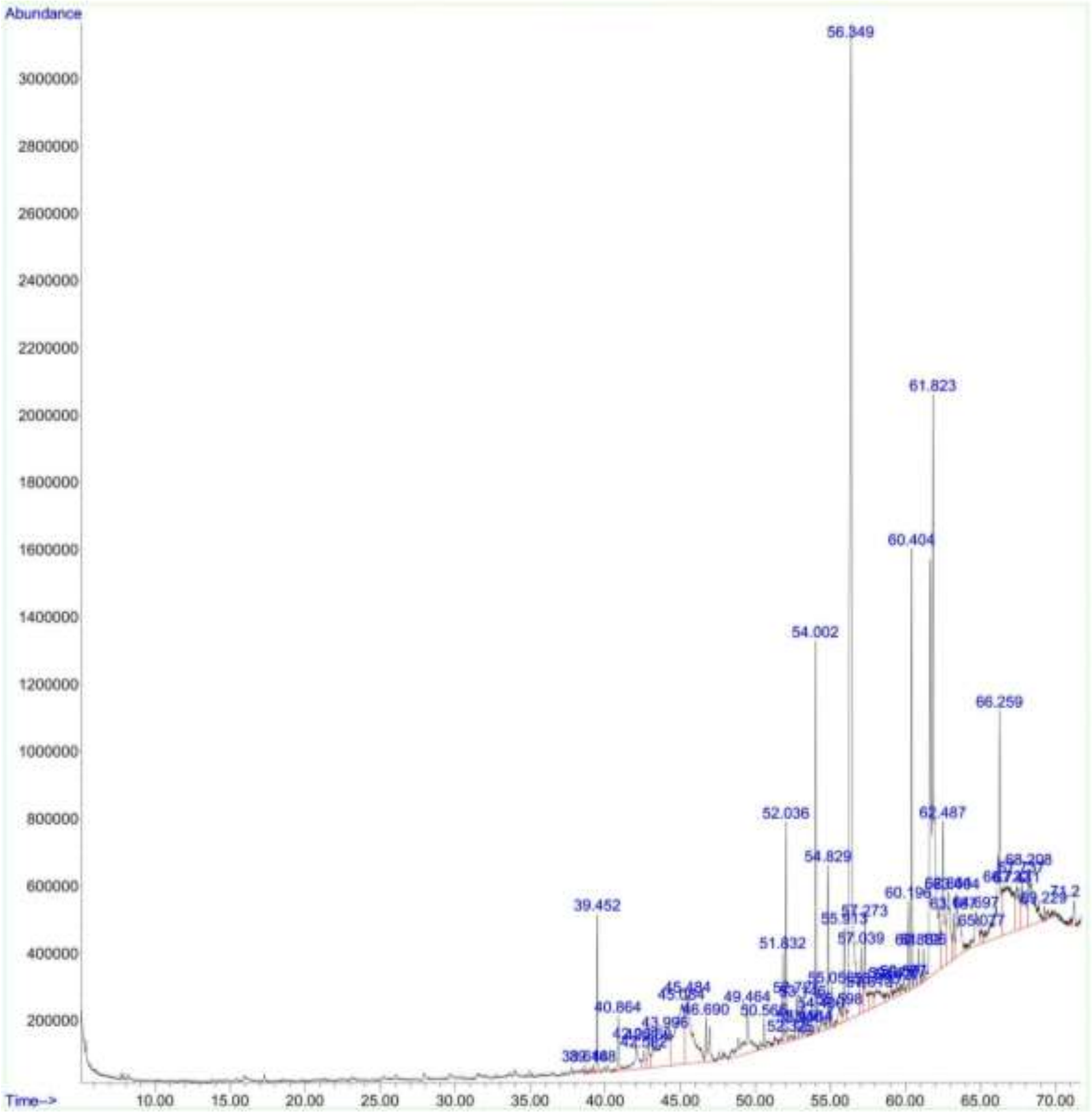

Figure S3: GC-MS profile of ethanomethanolic extract of *Strigosella africana* L. Botsch.

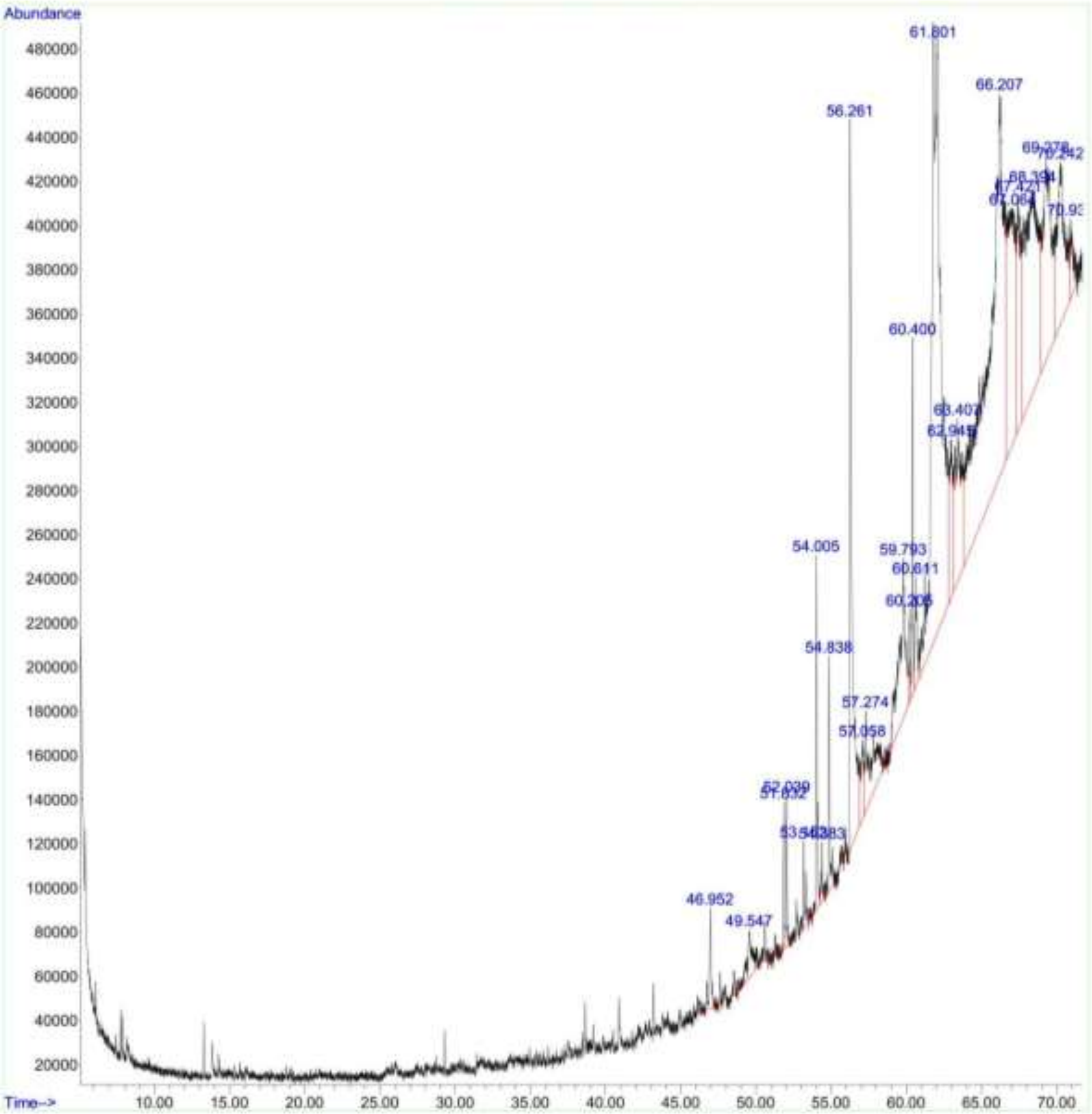

Figure S4: GC-MS profile of ethanomethanolic extract of *Chenopodium album* L. D: *Oxalis pes-caprae* L.

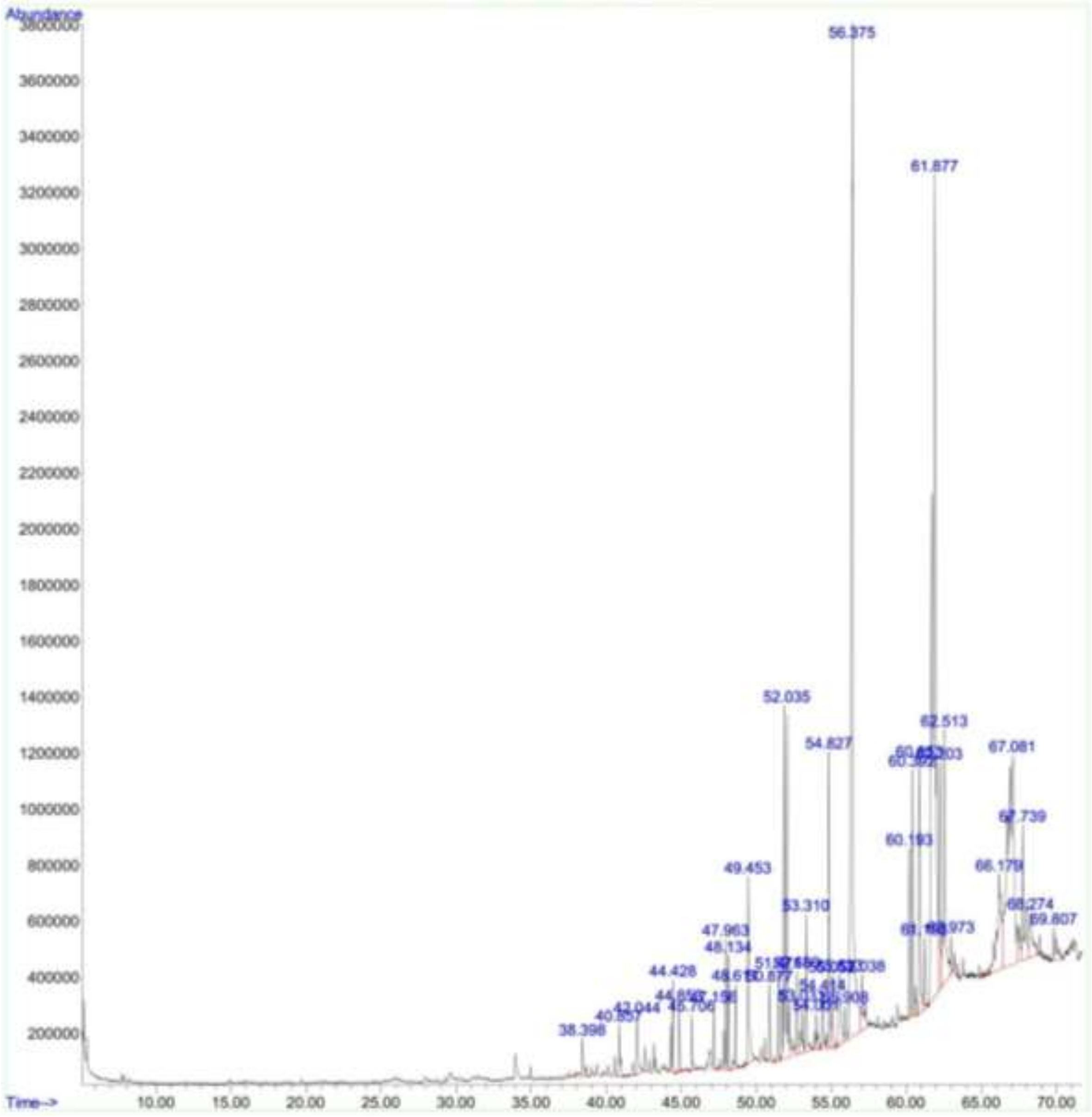

Figure S5: GC-MS profile of ethanomethanolic extract of *Oxalis pes-caprae* L.

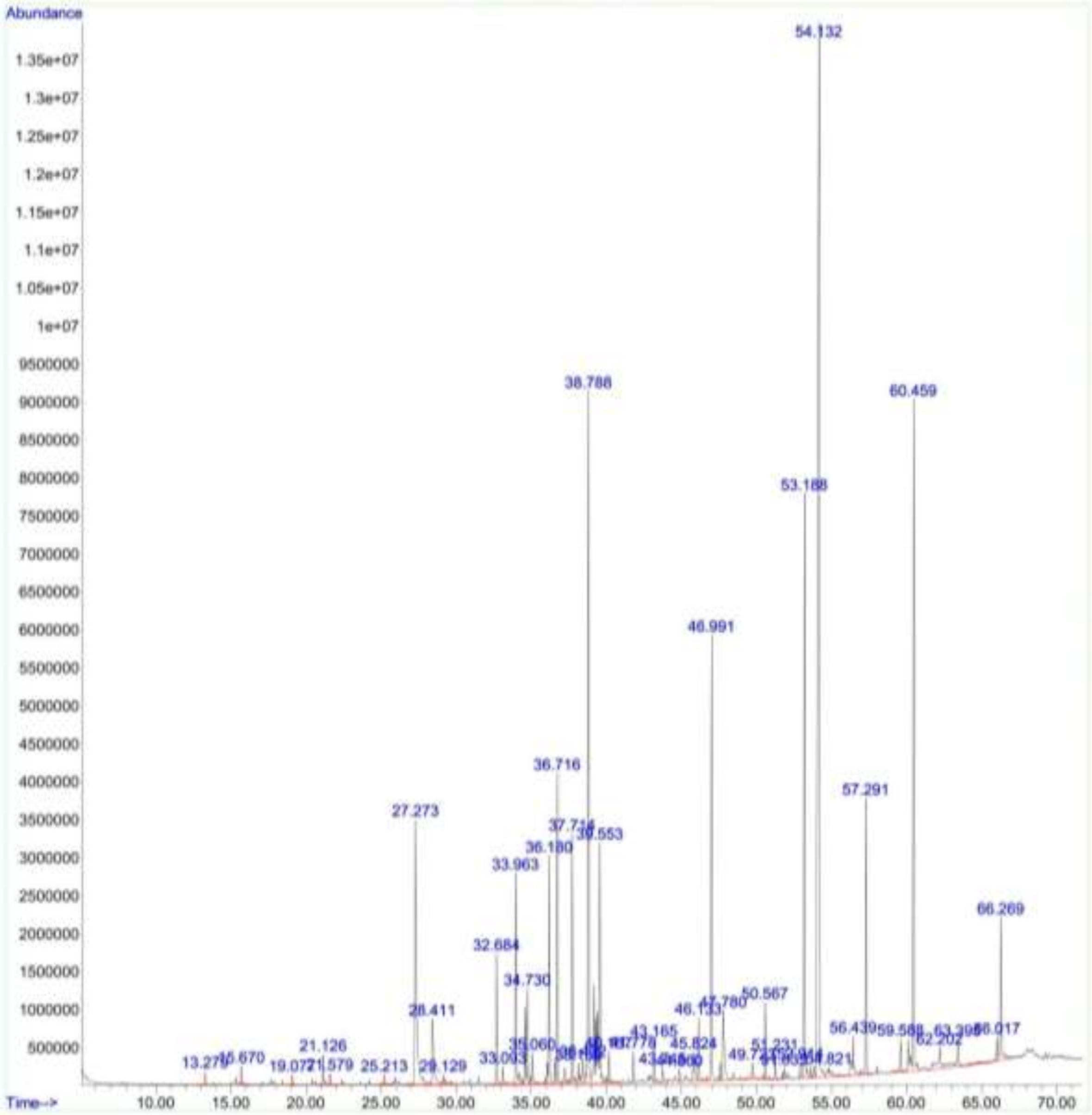

Figure S6: GC-MS profile of ethanomethanolic extract of *Ducrosia ismaelis* Asch.

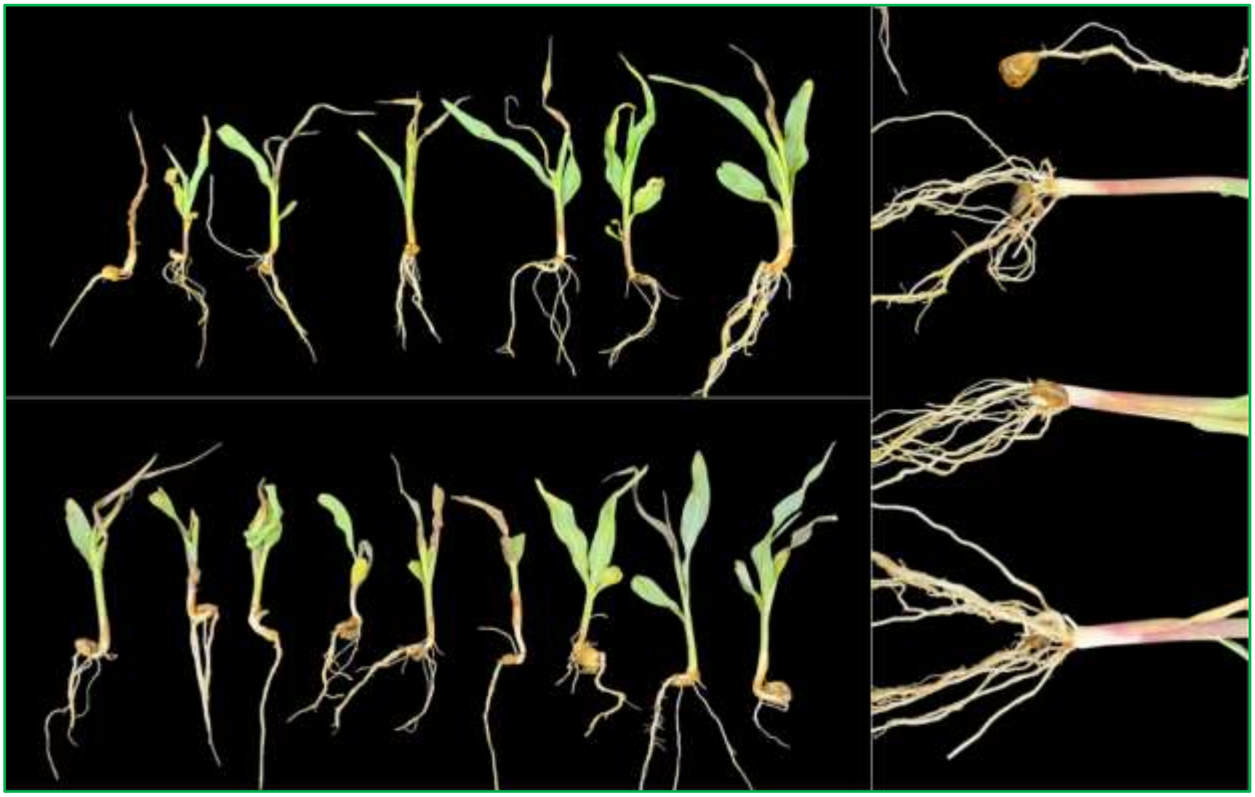

Figure S7: Sweet corn seedlings showing discoloration, browning, necrosis and wilting symptoms.

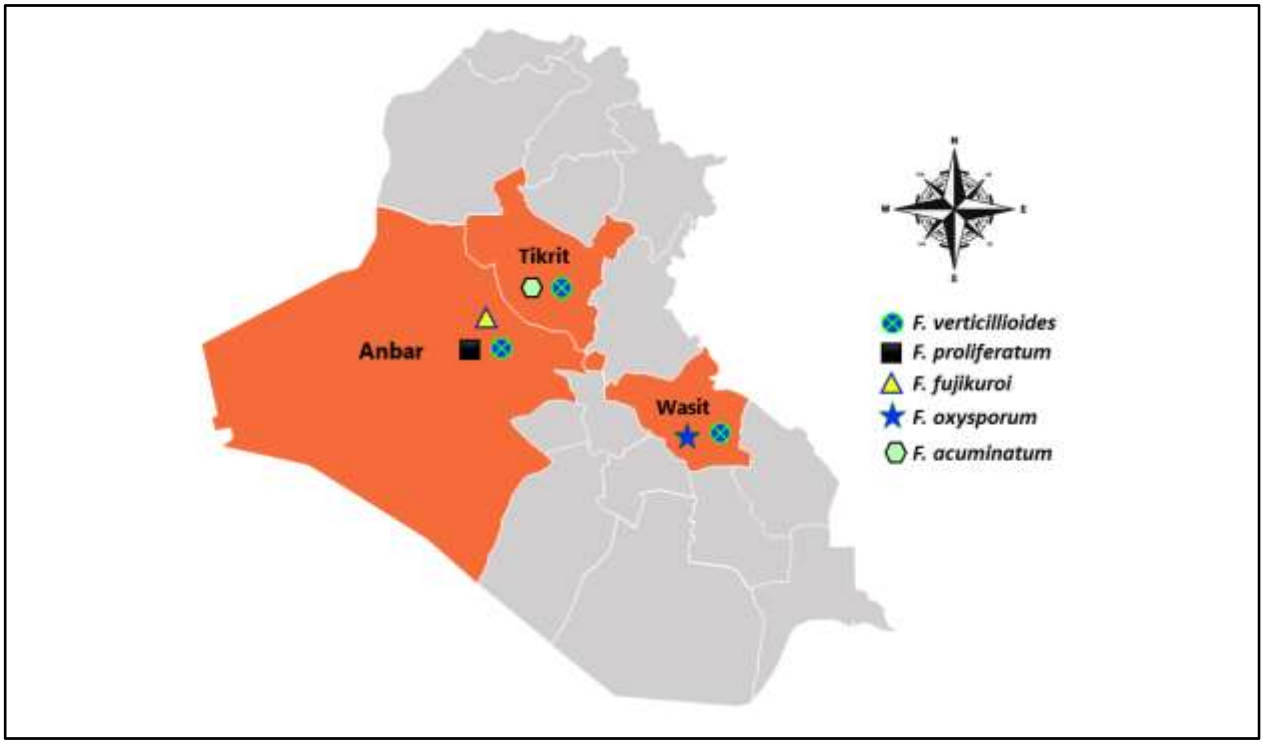

Figure S8: Map of Iraq showing sites of collected samples of sweet corn infected plants by *Gibberella fujikuroi* species complex.

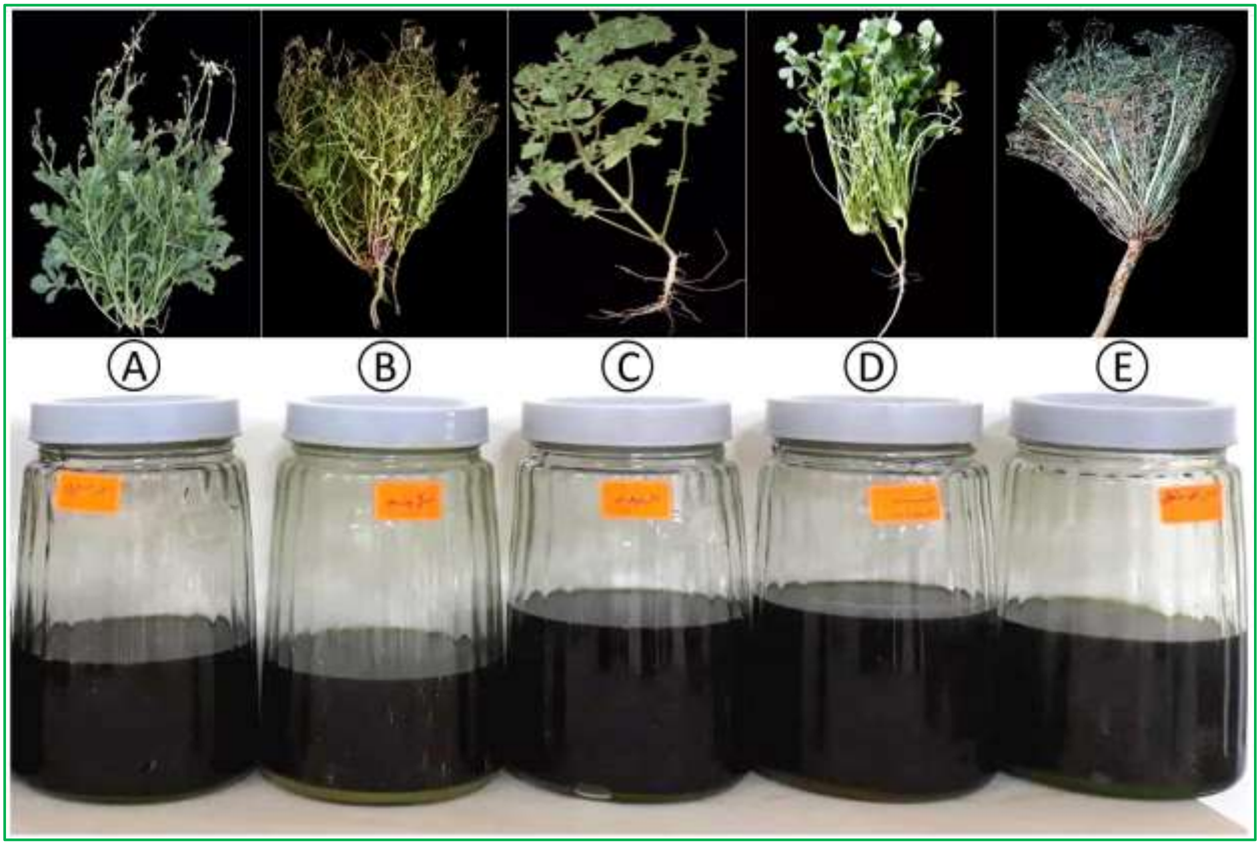

Figure S9: Ethanomethanolic extracts of the five investigated plant species. A: Arugula (*Eruca vesicaria* L. Cav.), B: African mustard (*Strigosella africana* L. Botsch.), C: White goosefoot (*Chenopodium album* L.), D: Bermuda buttercup (*Oxalis pes-caprae* L.), E: Ducrosia (*Ducrosia ismaelis* Asch.).
